# CDC6, a key replication licensing factor, is overexpressed and confers poor prognosis in diffuse large B-cell lymphoma

**DOI:** 10.1186/s12885-023-11186-6

**Published:** 2023-10-13

**Authors:** Mingfang Shen, Yunfeng Zhang, Lun Tang, Qinyan Fu, Jiawei Zhang, Yang Xu, Hui Zeng, Yuan Li

**Affiliations:** 1 Department of Hematology, the First Hospital of Jiaxing, 314001 Zhejiang, China; 2 Department of Hematology, the Second Affiliated Hospital, Zhejiang University School of Medicine, 310009 Zhejiang, China

**Keywords:** CDC6, Diffuse large B cell lymphoma, Cell cycle, Apoptosis, Prognosis

## Abstract

**Background:**

Cell division cycle 6 (CDC6) is a key licensing factor in the assembly of pre-replicative complexes at origins of replication. The role of CDC6 in the pathogenesis of in diffuse larger B-cell lymphoma (DLBCL) remains unknown. We aim to investigate the effects of CDC6 on the proliferation, apoptosis and cell cycle regulation in DLBCL cells, delineate its underlying mechanism, and to correlate CDC6 expression with clinical characteristics and prognosis of patients with DLBCL.

**Methods:**

Initial bioinformatic analysis was performed to screen the potential role of CDC6 in DLBCL. Lentiviral constructs harboring CDC6 or shCDC6 was transfected to overexpress or knockdown CDC6 in SUDHL4 and OCI-LY7 cells. The cell proliferation was evaluated by CCK-8 assay, cell apoptosis was detected by Annexin-V APC/7-AAD double staining, and cell cycle was measured by flow cytometry. Real time quantitative PCR and western blot was used to characterize CDC6 expression and its downstream signaling pathways. The clinical data of DLBCL patients were retrospectively reviewed, the CDC6 expression in DLBCL or lymph node reactive hyperplasia tissues was evaluated by immunohistochemistry.

**Results:**

In silico data suggest that CDC6 overexpression is associated with inferior prognosis of DLBCL. We found that CDC6 overexpression increased SUDHL4 or OCI-LY7 cell proliferation, while knockdown of CDC6 inhibited cell proliferation in a time-dependent manner. Upon overexpression, CDC6 reduced cells in G1 phase and did not affect cell apoptosis; CDC6 knockdown led to significant cell cycle arrest in G1 phase and increase in cell apoptosis. Western blot showed that CDC6 inhibited the expression of INK4, E-Cadherin and ATR, accompanied by increased Bcl-2 and deceased Bax expression. The CDC6 protein was overexpressed DLBCL compared with lymph node reactive hyperplasia, and CDC6 overexpression was associated with non-GCB subtype, and conferred poor PFS and OS in patients with DLBCL.

**Conclusion:**

CDC6 promotes cell proliferation and survival of DLBCL cells through regulation of G1/S cell cycle checkpoint and apoptosis. CDC6 is overexpressed and serves as a novel prognostic marker in DLBCL.

**Supplementary Information:**

The online version contains supplementary material available at 10.1186/s12885-023-11186-6.

## Introduction

Diffuse large B-cell lymphoma (DLBCL) is one of the most frequent lymphomas that accounts for 30–40% of non-Hodgkin lymphomas [1, 2]. With a frontline R-CHOP (rituximab, cyclophosphamide, doxorubicin, vincristine and prednisone) immunochemotherapy, the 5-year overall survival (OS) rate for DLBCL has increased to more than 60%, but 40–45% of DLBCL are still refractory or relapsed with poor prognosis [1, 2]. Risk stratification of DLBCL is mainly dependent on calculation of the International Prognostic Index (IPI), revised IPI or national comprehensive cancer network (NCCN)-IPI, primarily based on known clinical risk factors [3]. In addition, cell-of-origin (COO) defined by immunohistochemical (IHC) algorithms, are used as surrogates for gene expression profiling (GEP) to assign DLBCL as germinal center B-cell (GCB) or non-GCB subtype [4]. Generally, GCB DLBCL is associated with an improved outcome compared to non-GCB DLBCL in patients receiving the upfront standard of care [4]. Identification of novel biomarker may help to refine risk assessment, inform treatment decision-making and predict treatment response and clinical outcome.

DNA replication is an essential cellular process, in which cell division cycle 6 (CDC6) functions as a key licensing factor responsible for the assembly of pre-replicative complexes at origins of replication during the G1 phase of the cell cycle (Supplemental Fig. 1) [5, 6]. Previous studies have shown that CDC6 is a bona fide proto-oncogene that involves in tumor initiation and progression, and it is often over-expressed in many tumor types, providing the rationales for CDC6 as a novel therapeutic target [7, 8]. However, the clinical significance and mechanisms of action of CDC6 in lymphoma are poorly understood.

In this study, we investigated the role of CDC6 in the development of DLBCL, and explored the underlying mechanisms by which CDC6 regulates cell proliferation and apoptosis. To define the prognostic role in DLBCL, we retrospectively analyzed the clinical data of DLBCL patients, detected CDC6 expression by immunohistochemistry, and correlated CDC6 expression with clinicopathological features and prognosis of patients with DLBCL.

## Materials and methods

### Bioinformatic analysis

We used two series of DLBCL from 2 different databases: (1) The transcriptomic data of 47 DLBCL patients and 337 normal donors were obtained from The Cancer Genome Atlas (TCGA; https://cancergenome.nih.gov/) database and the Genotype-Tissue Expression (GTEX; https://gtexportal.org), respectively; (2) The CDC6 transcriptional expression and relevant survival information obtained from the Gene Expression Omnibus (GEO) (https://www.ncbi.nlm.nih.gov/geo/, accession number: GSE10846).

### Patients

Between January 2012 and December 2018, 60 patients with DLBCL were enrolled in the First Hospital of Jiaxing, Zhejiang, China. The pathological diagnosis was established or re-confirmed according to the 2016 WHO classification criteria for lymphohematopoietic tissues. Most patients were treated with the modified R-CHOP/CHOP 21 regimens (rituximab, cyclophosphamide, epirubicin or liposomal doxorubicin, vindesine, dexamethasone or prednisone), and a few elderly patients received reduced-dose R-CHOP or R-COP chemotherapy; for those did not achieve CR, the second-line regimens, such as GDP (gemcitabine, cisplatin, dexamethasone) and ICE (ifosfamide, carboplatin, etoposide, epirubicin), were given. In addition, 20 patients with reactive hyperplasia of lymph nodes were included the control group. The study was performed in accordance with the Declaration of Helsinki, and was approved by the institutional review board of the First Hospital of Jiaxing.

### Plasmids

To generate a construct overexpressing CDC6, cDNA fragment was amplified by the sense primer 5 ‘-TAGAGCTAGCGAAT**TCATGCCTCAAACCCGATCCC**-3’ and the antisense primer 5 ‘-CTTTGTAGTCGGATCC**AGGCAATCCAGTAGCTAAGAT**-3’, and cloned into pLenO-GTP-c-3flag vector, the resulting construct was designated as pLenO-CDC6. To generate construct expressing shRNA targeting CDC6, oligos were annealed and cloned into pLenR-GPH vector, the resulting construct was designated as pLenR-shCDC6. With the shRNA targeting region shown in bold, the oligos were sense 5 ‘-GATC**CCGGGCATTGAACAAAGCTAAA**CTTCCTGTCAGA**TTTAGCTTTGTTCAATGCCCG**TTTTTG-3’ and antisense 5 ‘- AATTCAAAAA**CGGGCATTGAACAAAGCTAAA**TCTGACAGGAAGTTTAGC**TTTGTTCAATGCCCGG**-3’.

### Cell culture and transfection

Human B-cell lymphoma cell line SUDHL4 was purchased from Cobioer Bioscience (Nanjing, China), OCI-LY7 was from Keygen Biotech (Nanjing, China). Both cell lines were cultured in RPMI-1640 with 10% FBS and antibiotics. To over-express or knockdown CDC6, SUDHL4 or OCI-LY7 was infected with lentivirus packaging with indicated plasmids, including pLenO vetor, pLenO-CDC6, pLenR-GPH vector and pLenR-GPH-shCDC6, respectively.

### Real time quantitative PCR

Total RNA was isolated by using Trizol reagents (Invitrogen) and reverse transcribed into cDNA. Quantitative real-time PCR was performed in triplicate, using One Step TB Green PrimeScript RT-PCR Kit II (SYBR Green) (TaKaRa, Japan) following the manufacturer’s instructions. The relative level of CDC6 mRNA was calculated using the method of 2^-ΔΔCt^ and normalized to human GAPDH gene. The primers for CDC6 included the upstream 5 ‘- GCAGTTCAATTCTGTGCCCG − 3’ and the downstream 5-ATAGCTCCTGCAAACATCCA-3 ‘; for GAPDH, the upstream primer was 5 ‘-AGATCATCAGCAATGCCTCCT-3’ and the downstream primer was 5 ‘-TGAGTCCTTCCACGATACCAA − 3’.

### Cell proliferation, apoptosis assays and cell cycle analysis

Cell proliferation was determined by using CCK-8 assay. SUDHL4 or OCI-LY7 cells were seeded at a density of 5 × 10^3^ per well in 96-well plates and 10 µl of CCK-8 reagent was added and incubated at 37 ° C for 3 h. The absorbance (OD) at 450 nm was measured. Cell apoptosis was determined by annexin V-APC/7-AAD apoptosis detection kit (Keygen Biotech, China), Briefly, after transfection for 24 h, 48 and 72 h, cells were collected, stained with annexin V-APC and 7-AAD according to the manufacturer’s instructions, and then analyzed by flow cytometry. Each experiment was done in triplicate. For DNA histogram analysis, cells were stained with propidium iodide, and analyzed by flow cytometry.

### Immunofluorescence

The transfected cells grown on chamber slides were fixed with 4% paraformaldehyde in phosphate-buffered saline (PBS) for 30 min at room temperature or overnight at 4℃, treated with 3% H_2_O_2_-methanol solution for 10 min, and washed with PBS. After blocking with goat serum for 20 min, cells were incubated with primary antibody against CDC6 (ab109315, abcam, UK) at 37℃ for 2 h, and then incubated with TRITC-conjugated secondary antibody (diluted 1:100) at 37℃ for 1 h. After washing, cell nuclei were stained with 4,6-diamidino-2-phenylindole (DAPI) for 5 min, and fluorescence was visualized using a confocal laser scanning microscope.

### Western blot

After 72-h transfection, cells were collected, washed twice with pre-cooled PBS, and lysed on ice with buffer containing 10 µl phosphatase inhibitor, 1 µl protease inhibitor, and 5 µl 100 mM PMSE for 30 min. The resulting cell lysate was centrifuged at 14,000 rpm for 15 min at 4℃, and the supernatant was aspirated for protein quantification by the Bradford method. Proteins were separated by SDS-PAGE electrophoresis, electro-transferred to a polyvinylidene fluoride (PVDF) membrane. After blocking at room temperature for 2 h, membrane was incubated with primary antibody overnight at 4℃, and then probed with secondary antibody at room temperature for 1–2 h. The blot was visualized by G: BOX chemiXR5 imaging and analyzed by Gel-Pro32 software. The primary antibodies against CDC6 (ab109315), caspase-3 (ab197202), bcl-2 (ab182858), Bax (ab182734), E-cadherin (ab40772), and ATR (ab13798) were purchased from abcam, UK; anti-INK4 (Cst 74,560) was from Cell Signaling Technology, US.

### Immunohistochemistry (IHC)

Specimens were fixed in 10% neutral buffered formalin solution, embedded in paraffin, serially sectioned at 4 μm, and then stained with CDC6 monoclonal antibody (ab109315) or antibodies such as anti-CD20, CD79a, CD3, CD5, CD10, BCL-6, MUM1, BCL-2, Ki-67, CyclinD1 by the EnVision Detection System K5007 (Dako, Denmark) according to the manufacturer’s instructions. Phosphate buffered saline (PBS) was used as a negative control. All specimens were reviewed and confirmed independently by two pathologists. Breast cancer tissue was used as CDC6 positive control. To quantity CDC6 expression, five high-power fields (x200) were randomly selected from each section, and 500 cells were counted and scored according to the immunoreactive score method. The presence of brownish-yellow granules in the nucleus or cytoplasm was recorded as CDC6 positive. Based on the percentage of positive cells, 0 point was assigned for < 25%, 1 point for 25-50%, 2 points for 50-75%, and 3 points for > 75%. The staining intensity was also categorized, with 1 point was assigned for no color, 2 points for light yellow, 3 points for brownish-yellow, and 4 points for dark brown. Both scores were multiplied to calculate a final score from 0 to 12, with < 6 being negative expression and ≥ 6 being positive expression.

### Statistical analysis

Overall survival (OS) was defined as the time from the day of diagnosis to death due to any cause or the last follow-up. Progression-free survival (PFS) was defined as the time from the day of diagnosis to disease recurrence, progression, or death from any cause or last follow-up. The Pearson correlation analysis was performed between CDC6 level and Myc or Ki67 expression using transcriptomic data. A *p* < 0.05 on both sides was considered statistically significant. Data were analyzed by SPSS 17.0 statistical software, enumeration data were expressed as percentage (%), and χ2 test was used for comparison; measurement data were expressed as mean ± standard deviation (sd), and one-way analysis of variance was used to analyze the OD value, apoptosis rate, cell cycle and CDC6 mRNA of SUDHL4; independent sample t-test was used to analyze the relationship between CDC6 protein expression and clinicopathological characteristics of DLBCL; Kaplan-Meier method was used for survival curve analysis, and univariate analysis and comparison of survival curves were performed through log-rank test.

## Results

### Bioinformatic analysis of CDC6 in DLBCL

**Fig. 1:**
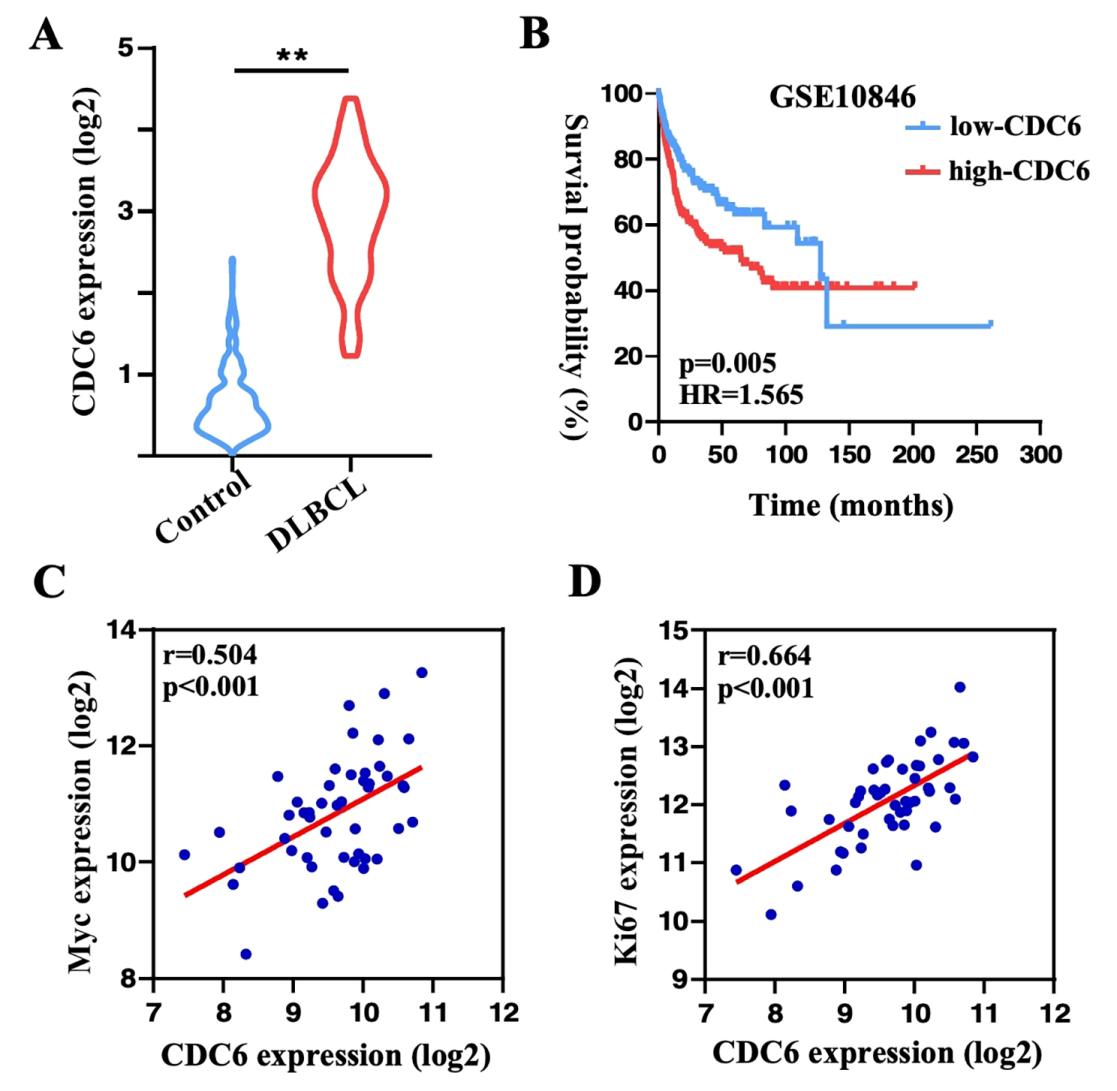
**Bioinformatic analysis shows that CDC6 is overexpressed in DLBCL. A**, CDC6 expression is significantly higher in DLBCL patients than in healthy donors (control). **: p < 0.001. **B**, the OS curve of DLBCL patients with the high- and low-CDC6 expression. C and D, in terms of expression level, CDC6 positively correlated with Myc (**C**) and Ki67(**D**), respectively

To explore the role of CDC6 in the DLBCL, we obtained transcriptomic data from TCGA and GTEX databases, and compared CDC6 expression levels between DLBCL patients and normal donors. We found that CDC6 was significantly upregulated in DLBCL compared with normal control (Fig. 1A). Interesting, using GSE10846 dataset from GEO database, we performed survival analysis and revealed that OS was significantly lower in DLBCL patients with high CDC6 expression than in those with low CDC6 expression (Fig. 1B). Moreover, there were positive correlations in expression levels between CDC6 and critical DLBCL-related markers, Myc and Ki67, respectively (Fig. 1C and D). Thus, in silico data suggest that CDC6 is overexpressed in DLBCL and is associated with inferior prognosis, which prompted us to investigate how CDC6 is involved in DLBCL pathogenesis.

### CDC6 expression and subcellular localization in B cell lymphoma cells

**Fig. 2:**
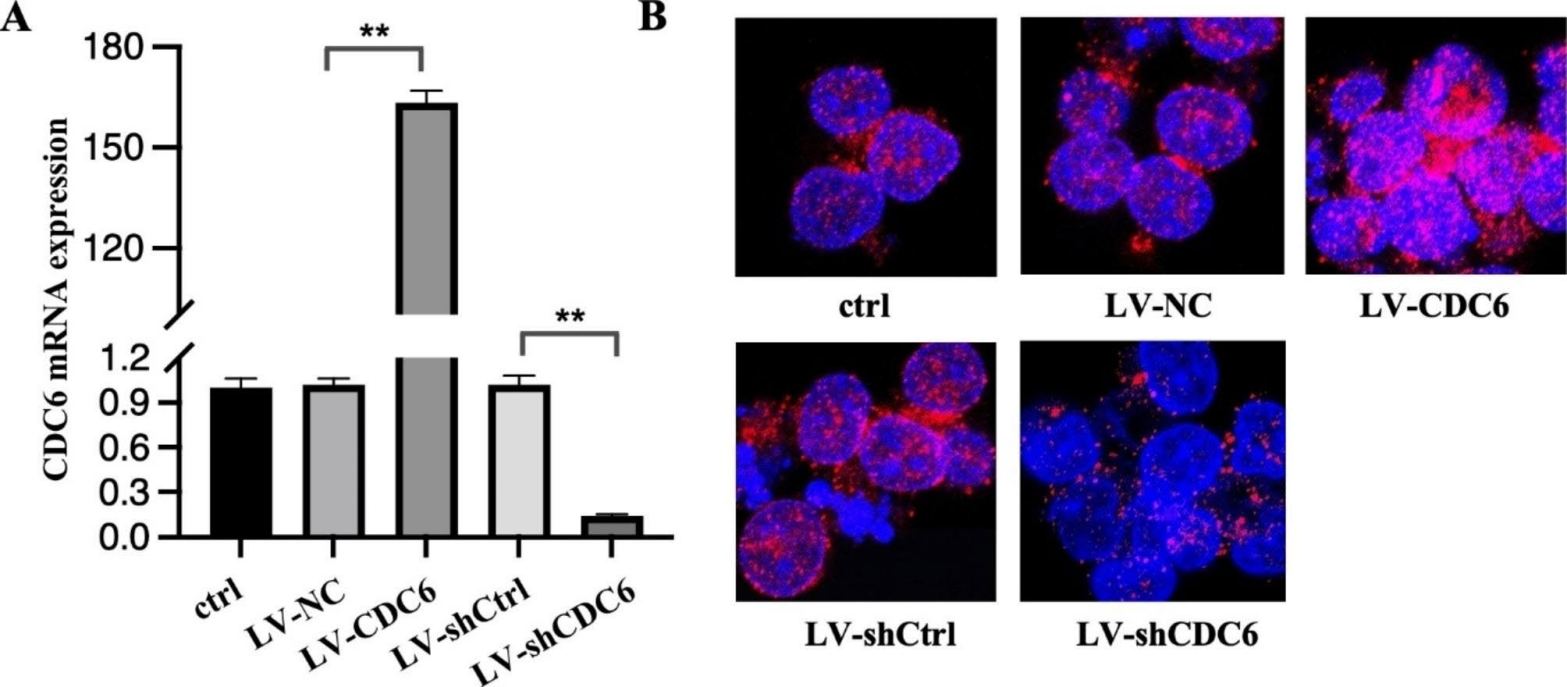
**CDC6 expression and subcellular localization in B cell lymphoma cells. A**, parental SUHDL4 cells (control) were transfected with pLenO vector (LV-NC), pLenO-CDC6 (LV-CDC6), pLenR-GPH vector (LV-shCtrl) and pLenR-shCDC6 (LV-shCDC6), respectively; 72 h after transfection, total RNAs were purified for real-time quantitative PCR. The relative expression of CDC6 was normalized to that of GAPDH. **B**, immunofluorescence was performed as described under “material and method” CDC6 is stained as red, nuclei as blue

To alter CDC6 expression in vitro, lentiviral construct harboring CDC6 or shCDC6 was transfected to overexpress or knockdown CDC6 in SUDHL4 cells, and real-time quantitative PCR was used to measure the CDC6 mRNA levels. As shown in Fig. 2A, when compared with the control, LV-NC and LV-shCtrl did not affect CDC6 expression; LV-CDC6 significantly upregulated CDC6 mRNA, P < 0.001, while LV-shCDC6 led to significant decrease in CDC6 mRNA, P < 0.001. Immunofluorescence analysis showed that CDC6 protein was localized in both nucleus and cytoplasm of SUDHL4 cells. When compared with LV-NC and LV-shCtrl cells, the fluorescence intensities were increased in CDC6-overexpressed cells (LV-CDC6), but decreased in CDC6-knockdown (LV-shCDC6) cells (Fig. 2B).

### CDC6 promotes B cell lymphoma cell proliferation

**Fig. 3:**
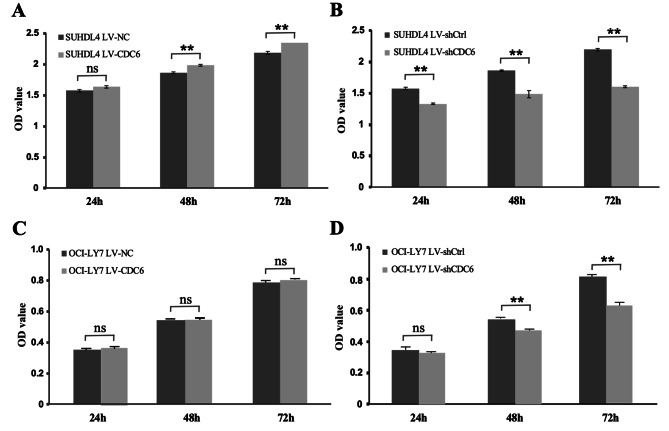
**CDC6 promotes B cell lymphoma cell proliferation. A** and **C**, SUHDL4 cells (**A**) and OCI-LY7 cells were transfected with pLenO vector (LV-NC) or pLenO-CDC6 (LV-CDC6) for 24, 48 and 72 h, respectively, and then subjected to CCK-8 assay. B and D, SUHDL4 cells (**B**) and OCI-LY7 (**D**) were transfected with pLenR vector (LV-shCtrl) or pLenR-shCDC6 (LV-shCDC6) for 24, 48 and 72 h, respectively, and followed by CCK-8 assay. ns, not significant; **, *p* < 0.05

To determine whether CDC6 affects BCL cell proliferation, CCK-8 assay was performed with or without alteration of CDC6 expression in SUDHL4 cells. We found that the SUDHL4 cells overexpressing CDC6 (LV-CDC6) had a significantly higher proliferation activity, which was shown as OD value, than control cells (LV-NC) (Fig. 3A). Conversely, knockdown of CDC6 inhibited SUCHL4 cell proliferation in a time-dependent manner, since LV-shCDC6 cells led to significant OD value reduction when compared with LV-shCtrl cells (Fig. 3B). To determine if the CDC6-dependent proliferation is cell line-specific, we tested another BCL cell line, OCI-LY7. As shown in Fig. 3C, CDC6 overexpression rarely promote OCI-LY7 proliferation, which is common due to high basal level of CDC6; however, when CDC6 was knocked down, OCI-LY7 cell proliferation was markedly inhibited (Fig. 3D). These data suggest that CDC6 can promote BCL cell proliferation.

### CDC6 regulates cell cycle progression and apoptosis in B cell lymphoma cells

**Fig. 4:**
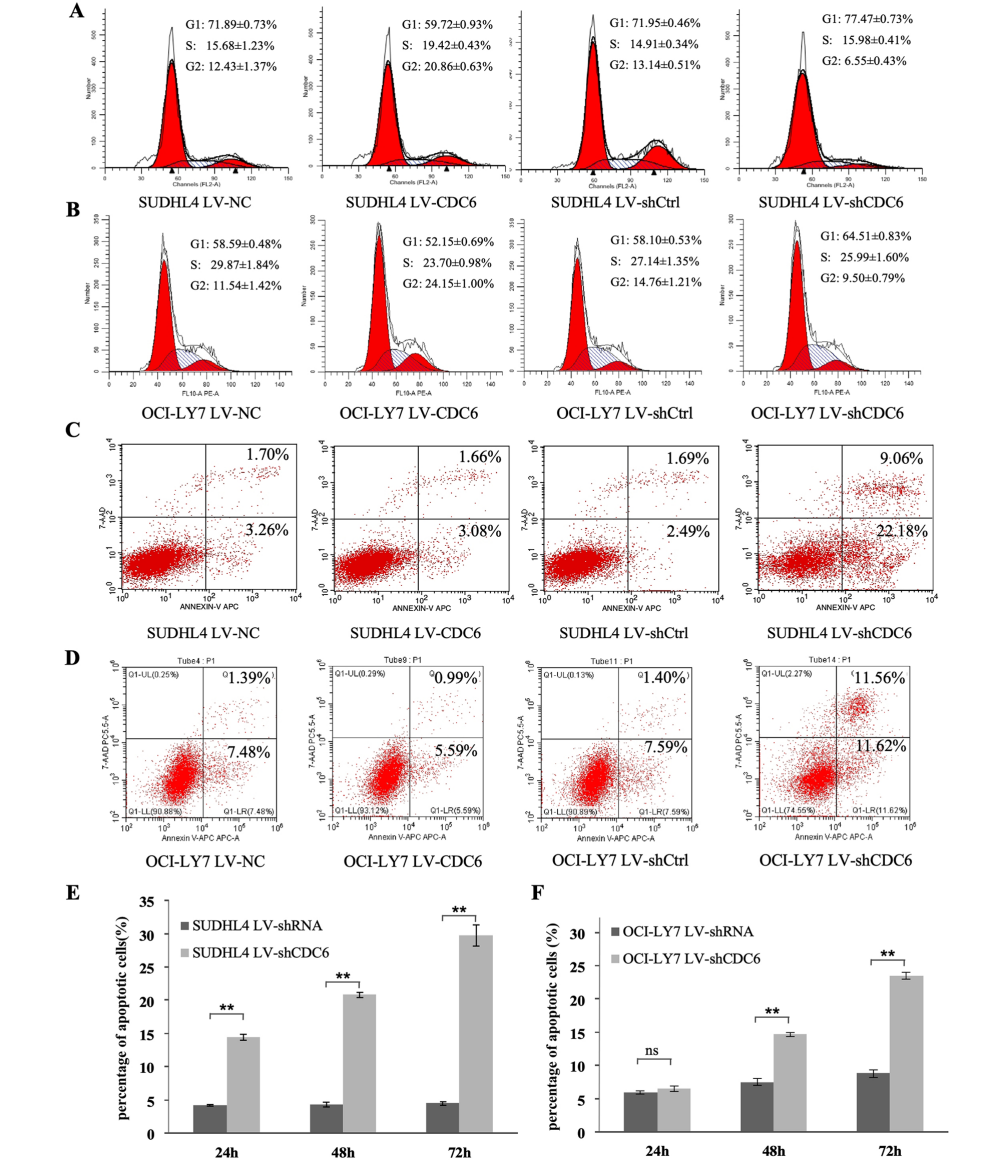
**CDC6 regulates cell cycle progression and apoptosis in B cell lymphoma cells.** A and B, SUHDL4 cells (**A**) and OCI-LY7 (**B**) were transfected with pLenO vector (LV-NC), pLenO-CDC6 (LV-CDC6), pLenR-GPH vector (LV-shCtrl) and pLenR-shCDC6 (LV-shCDC6) for 72 h, respectively, and cell cycle was analyzed by flow cytometry. **C** and **D**, cells were transfected as described in A and B, apoptosis was measured by flow cytometry following annexin V/7-AAD staining. E and F, SUHDL4 cells (**E**) and OCI-LY7 (**F**) cells transfected with pLenR vector (LV-shCtrl) or pLenO-shCDC6 (LV-shCDC6) for for 24, 48 and 72 h, respectively, and then apoptosis was evaluated. ns, not significant; **, *p* < 0.05

To test whether CDC6 affects cell proliferation through regulation of cell cycle and/or apoptosis, we performed flow cytometric analysis and annexin V/7-AAD staining on SUDHL4 and OCI-LY7 cells with and without CDC6 alterations. When compared with control cells (LV-NC), SUDHL4 cells overexpressing CDC6 (LV-CDC6) showed a significant decrease in the percentage of G1 phase,71.89% vs. 59.72%, *P* < 0.05, but increase in S (15.68% vs. 19.72%) and G2 (12.43% vs. 20.86%) phases, both *p* < 0.05. Likewise, knockdown of CDC6 (LV-shCDC6) led to a significant increase in the percentage of G1 phase (71.95% vs. 77.47%) and decrease in G2 phase (13.14% vs. 6.55%) compared with control (LV-shRNA), both p < 0.05(Fig. 4A). In OCI-LY7 cells, CDC6 overexpression reduces the percentage of G1 phase, 58.59% vs. 52.15%, *p* < 0.05; knockdown of CDC6 increases the percentage of G1 phase, 58.10% vs. 64.51, *p* < 0.05 (Fig. 4B). These data suggest that CDC6 regulates the G1/S checkpoint of the cell cycle.

Transfection of LV-NC, LV-ShCtrl or LV-CDC6 did not affect little if any cell apoptosis (Fig. 4C and D). When endogenous CDC6 was knocked down by LV-shCDC6 for 72 h, SUDHL4 cell apoptosis was increased from 3.18 to 31.24%, OCI-LY7 cell apoptosis increased from 8.99 to 23.18% (Fig. 4C and D); Furthermore, CDC6 knockdown induced significant cell apoptosis in a time-dependent manner, *p* < 0.05 (Fig. 4E F). Taken together, these data suggest that CDC6 is required for maintenance of BCL cell survival.

### The mechanisms underlying CDC6 regulated cell cycle and apoptosis

**Fig. 5:**
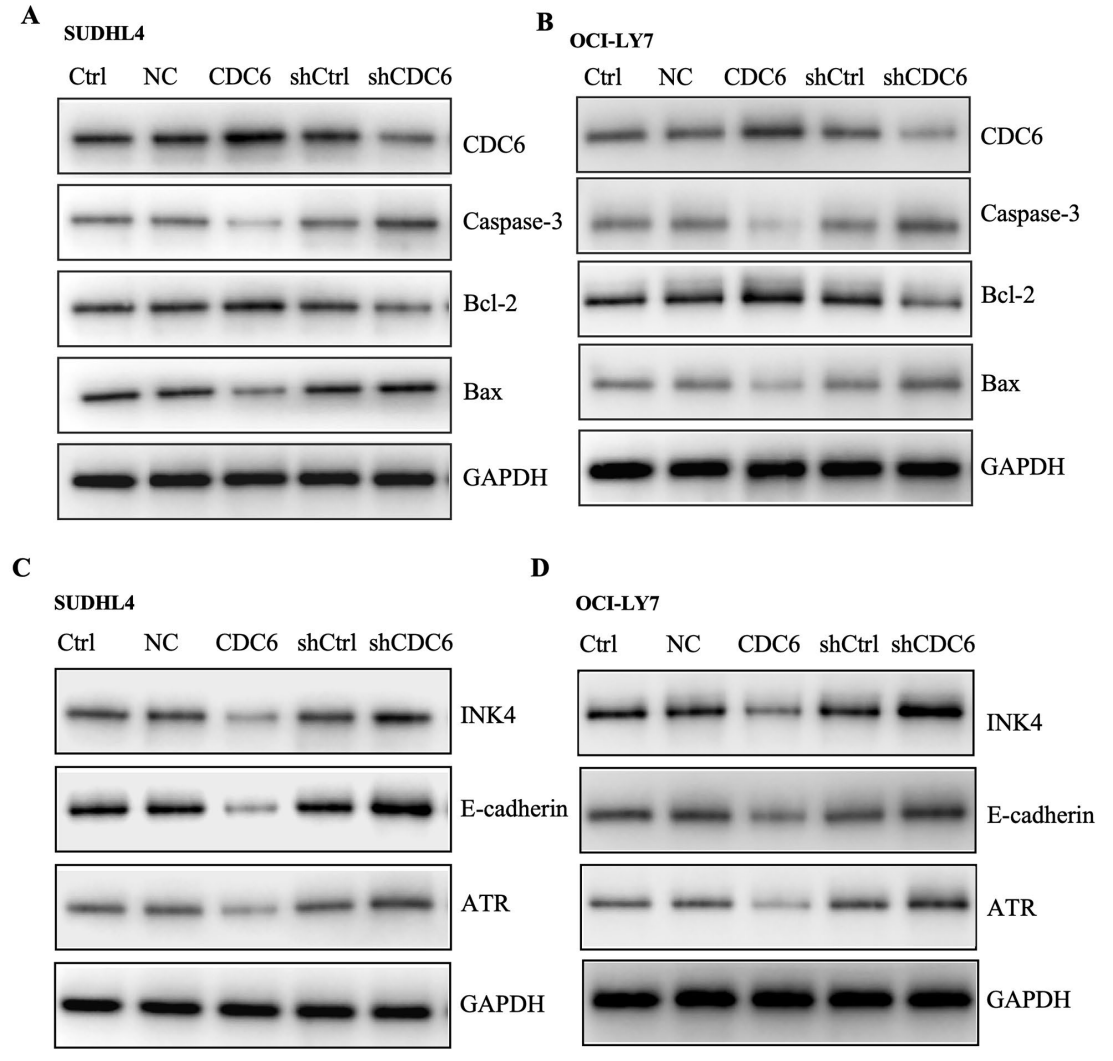
**CDC6 regulates downstream signalings related to cell cycle and apoptosis.** A and B, SUHDL4 cells (**A**) and OCI-LY7 (**B**) were transfected with pLenO vector (LV-NC), pLenO-CDC6 (LV-CDC6), pLenR-GPH vector (LV-shCtrl) and pLenR-shCDC6 (LV-shCDC6) for 72 h, respectively, and the levels of CDC6, caspase-3, Bcl-2, Bax and GAPDH were analyzed by Western blot analysis. C and D, the experiments were performed as described in **A** and **B**, the cell lysates were collected and analyzed by western blot with antibodies against INK4, E-Cadherin, ATR, and GAPDH, respectively

To investigate the molecular mechanisms by which CDC6 regulates cell survival, western blot was used to apoptosis-related factors including Bcl-2 family and caspase 3. We found that CDC6 overexpression induced Bcl-2 expression but inhibited the expression of Bax, a pro-apoptotic molecule within bcl-2 family, accompanied by a decrease in caspase-3; knockdown of CDC6 decreased Bcl-2 but increased Bax and caspase-3 expression. Thus, CDC6 upregulates Bcl-2 and downregulates Bax and caspase-3, resulting in attenuation of apoptosis. (Figure 5A and B). To further characterize the cell cycle signaling pathway, we detected the expression of tumor suppressor genes INK4 and E-Cadherin. As shown in Fig. 5C and D, CDC6 repressed INK4 and E-cadherin expression, accompanied by reduction of ATR, a kinase involving DNA damage response. In addition, the expression of INK4, E-Cadherin and ATR were increased when CDC6 was knocked down (Fig. 5C and D).

### CDC6 is overexpressed and confers poor prognosis in DLBCL

**Fig. 6:**
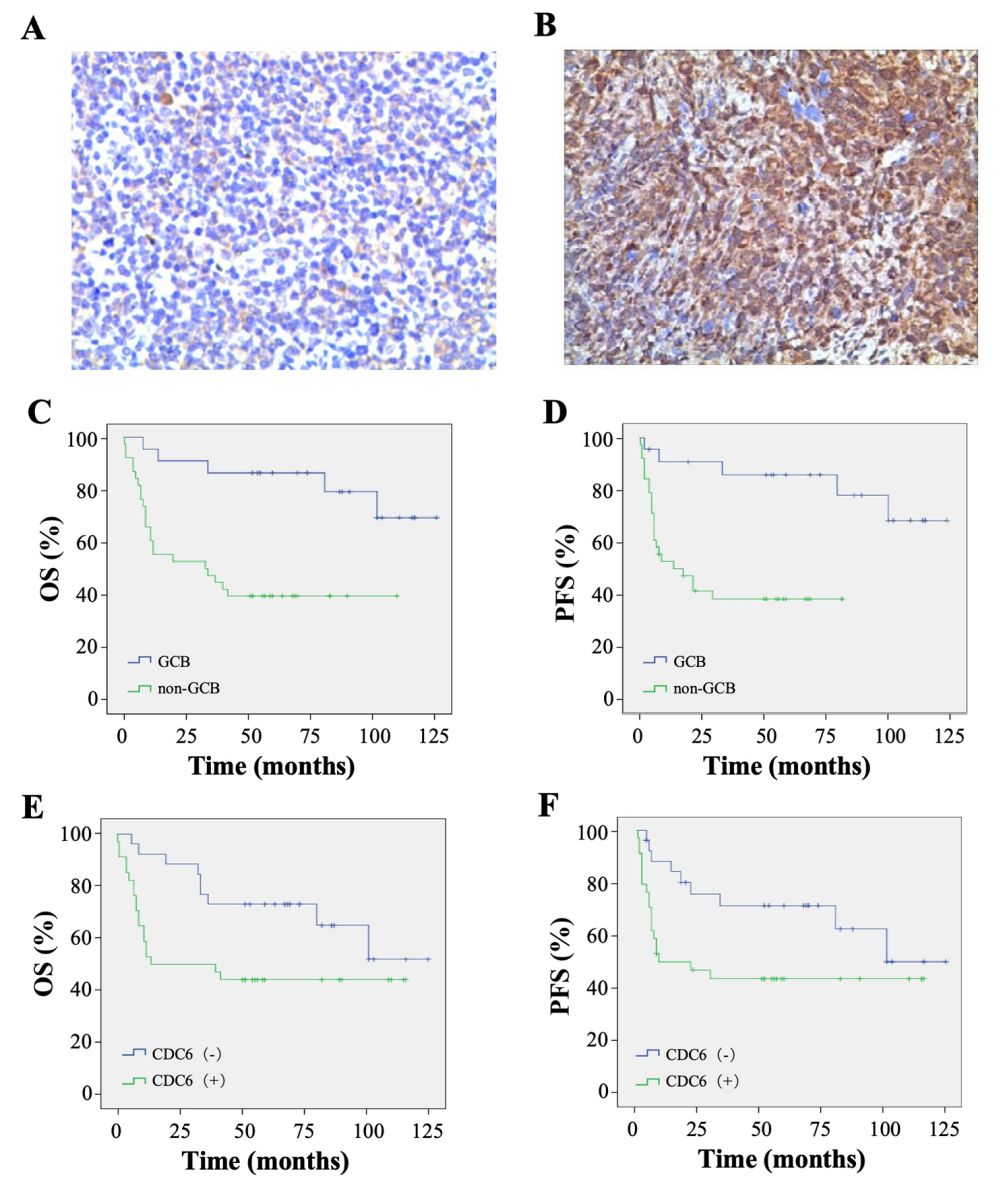
**CDC6 is overexpressed and confers poor prognosis in DLBCL.** A and B, Representative images of immunohistochemistry showing CDC6 expression in lymph node reactive hyperplasia (**A**) and DLBCL (**B**) tissues, EnVision method × 200. C and D, OS and PFS stratified by Hans classification. E and F, OS and PFS according to the status of CDC6 expression

To confirm the bioinformatic finding and determine the clinical significance of CDC6, CDC6 expression in DLBCL was compared with that in reactive hyperplasia of lymph nodes. CDC6 was stained as brownish-yellow granules that distribute in nucleus and cytoplasm of cells (Fig. 6A, B). The positive expression rate of CDC6 protein was 56.7% (34/60) in DLBCL and 25.0% (5/20) in lymph node reactive hyperplasia tissues, the difference of which was statistically significant, P = 0.014 (Table 1).

**Table 1:** CDC6 protein expression in DLBCL and reactive hyperplasia of lymph nodes

	Number (n)	CDC6 protein		χ^2^	*P*
		positive	negative		
DLBCL	60	34	26	6.020	0.014
lymph node reactive hyperplasia	20	5	15		

We next wanted to know which clinicopathological features are associated with CDC6 expression, the DLBCL patients’ characteristics and CDC6 expression are summarized in Table 2. Only Hans classification was associated with CDC6 expression, as non-GCB DLBCL showed markedly higher CDC6 expression than GCB DLBCL; while patients’ sex, age, LDH level, hemoglobin, Ann Arbor staging, IPI score and nodal involvement did not affect CDC6 expression.

**Table 2:** CDC6 expression and baseline clinical characteristics in the patients with DLBCL

clinical characteristics		cases (n)	CDC6 positivity No. (%)	*P* value
Sex	Male	35	17 (48.6)	0.134
	Female	25	17 (68.0)	
Age	≥ 60 years	42	26 (61.9)	0.211
	< 60 years	18	8 (44.4)	
LDH level	> 245 U/L	27	16 (59.3)	0.714
	≤ 245 U/L	33	18 (54.5)	
Hemoglobin	≥ 100 g/L	47	25 (53.2)	0.302
	< 100 g/L	13	9 (69.2)	
Ann Arbor Staging	I-II	32	17 (53.1)	0.554
	III-IV	28	17 (760.)	
IPI score	0–2	45	25 (55.6)	0.764
	3–5	15	9 (60.0)	
Hams classification	GCB	22	7 (31.8)	0.003
	Non-GCB	38	27 (71.1)	
Primary site	Nodal	28	14 (50.0)	0.330
	Extranodal	32	20 (62.5)	

To define the prognostic role of CDC6 in DLBCL, 60 patients with or without CDC6 expression were included for survival analysis. At a median follow up of 71 (range 39–131) months,46.7% (28/60) patients died, and mortality in CDC6-positive DLBCLs was higher than that in CDC6 negative DLBCLs, with deaths reported in 55.9% (19/34) and 34.6% (9/26) of patients, respectively, *P* = 0.102. Overall, the mean OS was 74.7 ± 7.2 months, and the mean PFS was 70.3 ± 7.6 months. As expected, patients with GCB subtype had superior OS and PFS than those with non-GCB subtype, 105.8 ± 8.3 vs. 51.9 ± 7.8 months (*P* = 0.001) and 104.3 ± 8.9 vs. 36.7 ± 6.1 months (*P* = 0.001), respectively (Fig. 6C, D). When compared with CDC6-negative DLBCL, CDC6-positive DLBCL resulted in significantly poorer OS and PFS, 57.7 ± 9.2 vs. 92.0 ± 9.1 months (*P* < 0.05) and 54.6 ± 9.5 vs. 87.1 ± 10.3 months (*P* < 0.05), respectively (Fig. 6E, F).

## Discussion

DNA replication is a fundamental biological process that is tightly coordinated with cell division to maintain genomic integrity. Dysfunction of DNA replication machinery results in genomic instability, which make normal cells vulnerable to malignant transformation [9]. CDC6 is an essential regulator of DNA replication in eukaryotic cells [10, 11]. It mainly involves in the assembly of the “pre-replication complex (pre-RC)” during G1 phase, and also regulate G2/M checkpoint through Chk1 activation, thereby ensuring proper cell cycle progression [12, 13]. Thus, aberrant CDC6 expression will compromise normal DNA replication and cause cell cycle dysregulation, which is closely related to tumorigenesis [14, 15]. For instance, CDC6 overexpression enables repeated DNA replication, resulting in genomic instability and oncogene activations [16, 17]. CDC6 activates G2/M phase replication checkpoint through CDC6-ATR-Chk1 signaling to promote the survival of cancer cells [18, 19]. CDC6 is indispensable for KRAS-induced cell transformation [20].

DLBCL is an aggressive lymphoid malignancy characterized by highly heterogeneous pathological features and clinical outcomes, integration of novel biomarkers will provide opportunity for individualized or precision treatment. In this study, for the first time we found that CDC6 expression was increased in DLBCL compared with reactive lymphocytic hyperplasia, CDC6 overexpression was often seen in non-GCB subtype and predicted adverse prognosis in patients with DLBCL. It has shown that CDC6 is overexpressed in several cancers such as lung [21], breast [8], ovarian [22] and prostate cancers [23] as well as chronic myelogenous leukemia [24]. CDC6 overexpression can result from gene amplification, E2F1/2 upregulation [15, 21] and alternative splicing, the latter of which predominantly generates a short CDC6 isoform resistant to degradation due to the loss of microRNA(miRNA)-binding sites in its 3’untranslated regions [25]. Interestingly, Cdc6 G1321A polymorphism (V441I, rs13706) was found to affect the risk of B cell lymphoma, and the AG or combined AA/AG genotype was associated with decreased risk for DLBCL, possibly through modulating mRNA secondary structure [26]. Therefore, it is necessary to further investigate the mechanisms underlying CDC6 overexpression and whether gene polymorphism affects CDC6 protein expression in DLBCL.

Given the important role CDC6 play in DLBCL pathogenesis, we provide preliminary evidence supporting CDC6 as a potential therapeutic target. In SUDHL4 cells, CDC6 knockdown induces cell cycle arrest and apoptosis, which is consistent with previous studies in other cancer types [27–29]. In this study, we found that after CDC6 overexpression, ATR was down-regulated, and cell cycle showed a decrease G1 phase and an increase in G2 phase. ATR protein, a kinase involving in cell cycle regulation, was up-regulated upon CDC6 knockdown, leading to cell cycle arrest in G1 phase. When CDC6 inhibition persisted longer, pro-apoptotic Bax increased, anti-apoptotic Bcl-2 decreased, and caspase 3 was activated to induce cell apoptosis of SUDHL4 cells. A variety of CDC6 downstream signaling pathways contribute to the dysregulation of cell cycle and proliferation in cancer [30]. CDC6 regulate cell cycle transition from G1 to S phase through prompting ATR to bind to chromatin, activating cell cycle checkpoints [31, 32]; meanwhile, CDC6 is also a key regulator of G2/M checkpoint, and overexpression of CDC6 in G2 phase can prevent cell cycle progression to M phase by activating Chk1 [33–35]. Consistent with previous findings [28, 36], we found that, INK4 and E-cadherin were down-regulated when CDC6 was overexpressed, while increased after CDC6 knockdown, suggesting that CDC6 promotes lymphomagenesis through transcriptional repression of tumor suppressor genes. Feng et al. [27] demonstrated that knockdown of CDC6 led to tumor cell apoptosis, but did not affect normal cells, regardless of p53 mutation in cancer [34]. Karanika et al. [19] reported that silencing CDC6 synergized with Chk1/2 inhibitor to kill prostate cancer cells. It is tempting to test if targeting CDC6 could overcome the adverse impact of p53 mutation in DLBCL, and if CDC6 inhibition could synergize with CHK1/2 inhibitor in DLBCL treatment.

In summary, we found that CDC6 is often overexpressed in DLBCL, and it may play a role in the development of DLBCL and serves as an adverse prognosis factor. CDC6 promotes lymphoma cell survival and prevents cell apoptosis, possibly through cell cycle regulation, Bcl2 upregulation and downregulation of Bax and INK4 and E-cadherin. Our data support CDC6 as a potential novel target for B cell lymphoma treatment, which needs to be further explored.

### Electronic supplementary material

Below is the link to the electronic supplementary material.

Supplementary Material 1

Supplementary Material 2

Supplementary Material 3

Supplementary Material 4

Supplementary Material 5

Supplementary Material 6

Supplementary Material 7

## Data Availability

The datasets generated and/or analyzed during the current study are included in this published article and its supplementary information files. We used two series of DLBCL from 2 different databases: (1) The transcriptomic data of 47 DLBCL patients and 337 normal donors were obtained from The Cancer Genome Atlas (TCGA; https://cancergenome.nih.gov/) database and the Genotype-Tissue Expression (GTEX; https://gtexportal.org), respectively; (2) The CDC6 transcriptional expression and relevant survival information obtained from the Gene Expression Omnibus (GEO) (https://www.ncbi.nlm.nih.gov/geo/, accession number: GSE10846).
