# Supplementary figures and images for: CDC6, a key replication licensing factor, is overexpressed and confers poor prognosis in diffuse large B-cell lymphoma
Source: BMC Cancer. 2023 Oct 13;23:978. doi: 10.1186/s12885-023-11186-6 (PMC10571299; doi:10.1186/s12885-023-11186-6)

CDC6 immunofluorescence

LV-CDC6


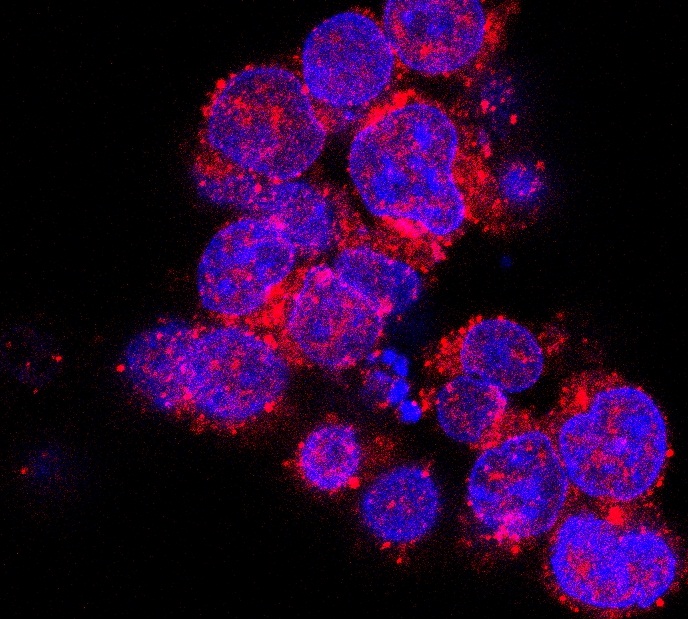


Control


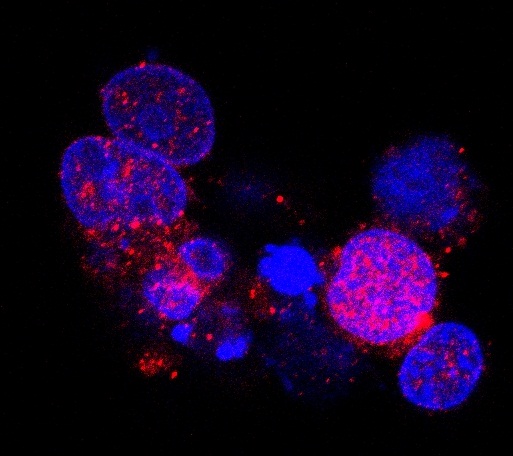


LV-NC


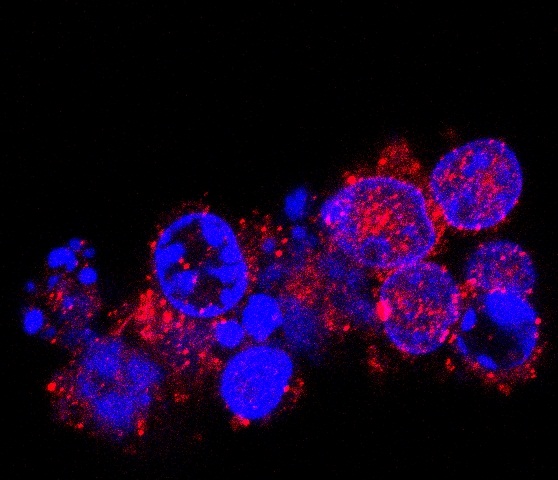


LV-shRNA
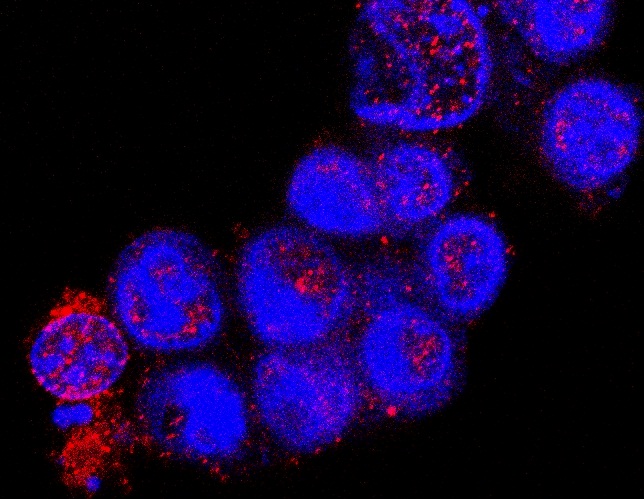


LV-shCDC6


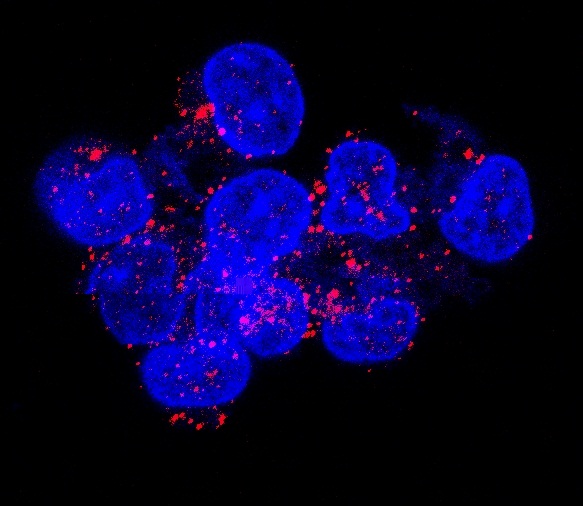

Supplement: Supplementary file 2 — Supplementary Material 2 [file 12885_2023_11186_MOESM2_ESM.docx]
