# Supplementary material for: CDC6, a key replication licensing factor, is overexpressed and confers poor prognosis in diffuse large B-cell lymphoma
Source: BMC Cancer. 2023 Oct 13;23:978. doi: 10.1186/s12885-023-11186-6 (PMC10571299; doi:10.1186/s12885-023-11186-6)
Supplement: Supplementary file 3 — Supplementary Material 3 [file 12885_2023_11186_MOESM3_ESM.docx]

| **SUDHL4 24 h CCK8 assay** | | | | | |
| --- | --- | --- | --- | --- | --- |
| group | parental | LV-NC | LV-CDC6 | LV-shCtrl | LV-shCDC6 |
| OD | 1.6178 | 1.555 | 1.5978 | 1.5262 | 1.3216 |
|  | 1.5907 | 1.5748 | 1.6384 | 1.5707 | 1.3101 |
|  | 1.6304 | 1.5569 | 1.5921 | 1.5636 | 1.2871 |
|  | 1.5883 | 1.6129 | 1.6713 | 1.5597 | 1.361 |
|  | 1.6267 | 1.5629 | 1.6757 | 1.6386 | 1.3605 |
| mean | 1.611 | 1.573 | 1.635 | 1.572 | 1.328 |
| SD | 0.020 | 0.024 | 0.039 | 0.041 | 0.032 |
| inhibition% | | 2.38% | -1.51% | 2.42% | 17.55% |
|  |  |  |  |  |  |
| **SUDHL4 48 h CCK8 assay** | | | | | |
| group | parental | LV-NC | LV-CDC6 | LV-shCtrl | LV-shCDC6 |
| OD | 1.9308 | 1.9019 | 2.0254 | 1.8643 | 1.4336 |
|  | 1.8688 | 1.8826 | 1.9901 | 1.8939 | 1.5717 |
|  | 1.9707 | 1.9184 | 1.9743 | 1.8564 | 1.5067 |
|  | 1.9304 | 1.7967 | 1.9371 | 1.8561 | 1.4444 |
|  | 1.9927 | 1.8139 | 2.0068 | 1.8169 | 1.4573 |
| mean | 1.939 | 1.863 | 1.987 | 1.858 | 1.483 |
| SD | 0.047 | 0.054 | 0.034 | 0.027 | 0.057 |
| inhibition% | | 3.92% | -2.48% | 4.19% | 23.52% |
|  |  |  |  |  |  |
| **SUDHL4 72 h CCK8 assay** | | | | | |
| group | parental | LV-NC | LV-CDC6 | LV-shCtrl | LV-shCDC6 |
| OD | 2.3138 | 2.2012 | 2.2769 | 2.1728 | 1.6223 |
|  | 2.239 | 2.1838 | 2.3317 | 2.1909 | 1.5764 |
|  | 2.247 | 2.1658 | 2.3475 | 2.1502 | 1.5492 |
|  | 2.3074 | 2.2185 | 2.3447 | 2.1761 | 1.592 |
|  | 2.3163 | 2.1658 | 2.4204 | 2.2746 | 1.6577 |
| mean | 2.285 | 2.187 | 2.344 | 2.193 | 1.600 |
| SD | 0.038 | 0.023 | 0.051 | 0.048 | 0.042 |
| inhibition% | | 4.28% | -2.61% | 4.02% | 29.99% |

| **OCI-LY7 24h CCK8 assay** | | | | | | |
| --- | --- | --- | --- | --- | --- | --- |
| group | parental | LV-NC | LV-CDC6 | | LV-shCtrl | LV-shCDC6 |
| OD | 0.3613 | 0.3494 | 0.3348 | | 0.3445 | 0.3248 |
|  | 0.3585 | 0.3556 | 0.3506 | | 0.3309 | 0.3409 |
|  | 0.3592 | 0.3359 | 0.3677 | | 0.3662 | 0.3381 |
| mean | 0.3597 | 0.3470 | 0.3510 | | 0.3472 | 0.3346 |
| SD | 0.0015 | 0.0101 | 0.0165 | | 0.0178 | 0.0086 |
| inbitition% | 0.00% | 3.53% | 2.40% | | 3.47% | 6.97% |
|  |  |  |  | |  |  |
| **OCI-LY7 48h CCK8 assay** | | | | | | |
| group | parental | LV-NC | | LV-CDC6 | LV-shCtrl | LV-shCDC6 |
| OD | 0.5537 | 0.5442 | | 0.5402 | 0.5348 | 0.4571 |
|  | 0.5588 | 0.5401 | | 0.5636 | 0.5485 | 0.4803 |
|  | 0.5620 | 0.5390 | | 0.5458 | 0.5425 | 0.4655 |
| mean | 0.5582 | 0.5411 | | 0.5499 | 0.5419 | 0.4676 |
| SD | 0.0042 | 0.0027 | | 0.0122 | 0.0069 | 0.0117 |
| inbitition% | 0.00% | 3.06% | | 1.49% | 2.91% | 16.22% |
|  |  |  | |  |  |  |
| **OCI-LY7 72h CCK8 assay** | | | | | | |
| group | parental | LV-NC | | LV-CDC6 | LV-shCtrl | LV-shCDC6 |
| OD | 0.8206 | 0.7829 | | 0.8025 | 0.7946 | 0.6213 |
|  | 0.8141 | 0.7638 | | 0.8083 | 0.7843 | 0.5924 |
|  | 0.8157 | 0.7931 | | 0.7944 | 0.8092 | 0.6386 |
| mean | 0.8168 | 0.7799 | | 0.8017 | 0.7960 | 0.6174 |
| SD | 0.0034 | 0.0149 | | 0.0070 | 0.0125 | 0.0233 |
| inbitition% | 0.00% | 4.51% | | 1.84% | 2.54% | 24.41% |

Note: Cell proliferation was determined by using CCK-8 assay. SUHDL4 cells and OCI-LY7 cells were transfected with pLenO vector (LV-NC), pLenO-CDC6 (LV-CDC6), pLenR vector (LV-shCtrl) or pLenR-shCDC6 (LV-shCDC6) for 24, 48 and 72 h, respectively, and then subjected to CCK-8 assay. Cells were seeded at a density of 5 × 10^3^ per well in 96-well plates and 10 μl of CCK-8 reagent was added and incubated at 37 ° C for 3 h. The absorbance (OD) at 450 nm was measured.
