# Supplementary material for: CDC6, a key replication licensing factor, is overexpressed and confers poor prognosis in diffuse large B-cell lymphoma
Source: BMC Cancer. 2023 Oct 13;23:978. doi: 10.1186/s12885-023-11186-6 (PMC10571299; doi:10.1186/s12885-023-11186-6)
Supplement: Supplementary file 4 — Supplementary Material 4 [file 12885_2023_11186_MOESM4_ESM.docx]

**The followings are from experiments using SUDHL4 cells**


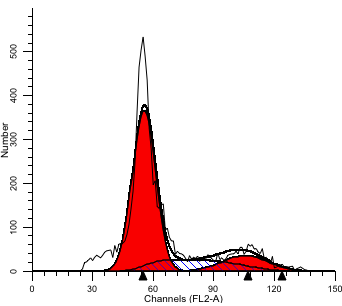


File analyzed: Data.001

Diploid: 100.00 %

Dip G1: 70.44 % at 55.65

Dip G2: 12.81 % at 105.74

Dip S: 16.74 % G2/G1: 1.90

%CV: 10.20


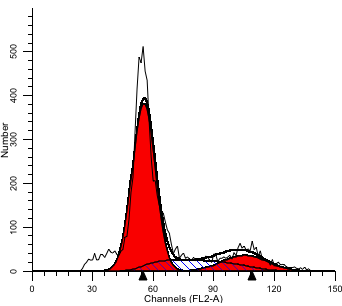


File analyzed: Data.002

Diploid: 100.00 %

Dip G1: 71.38 % at 55.50

Dip G2: 12.60 % at 105.46

Dip S: 16.02 % G2/G1: 1.90

%CV: 10.07


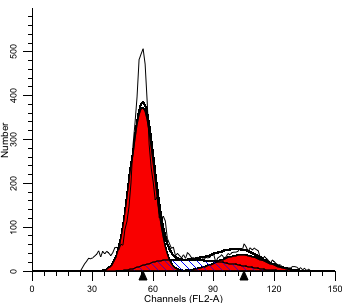


File analyzed: Data.

003Diploid: 100.00 %

Dip G1: 70.87 % at 54.79

Dip G2: 13.25 % at 104.11

Dip S: 15.87 % G2/G1: 1.90

%CV: 10.55

Parental cells


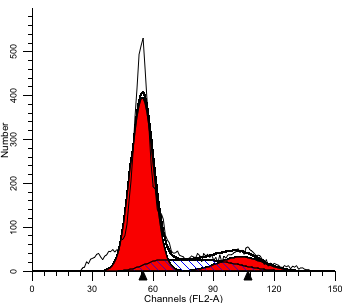


File analyzed: Data.004

Diploid: 100.00 %

Dip G1: 71.92 % at 54.66

Dip G2: 10.99 % at 103.85

Dip S: 17.09 % G2/G1: 1.90

%CV: 10.05


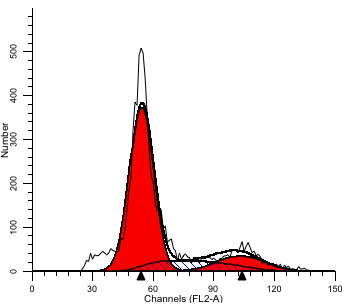


File analyzed: Data.005

Diploid: 100.00 %

Dip G1: 72.60 % at 54.44

Dip G2: 12.56 % at 103.44

Dip S: 14.84 % G2/G1: 1.90

%CV: 10.79


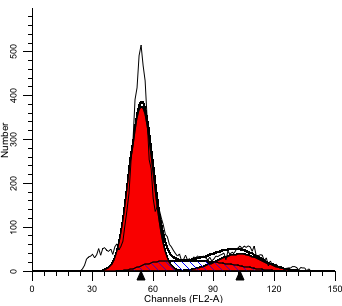


File analyzed: Data.006

Diploid: 100.00 %

Dip G1: 71.15 % at 54.23

Dip G2: 13.73 % at 103.05

Dip S: 15.12 % G2/G1: 1.90

%CV: 10.53

SUDHL4 LV-NC


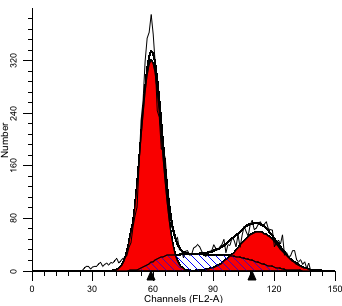


File analyzed: Data.007

Diploid: 100.00 %

Dip G1: 59.86 % at 59.12

Dip G2: 21.05 % at 112.34

Dip S: 19.09 % G2/G1: 1.90

%CV: 8.97


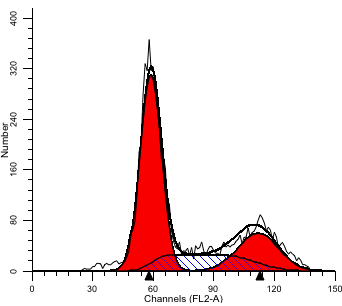


File analyzed: Data.008

Diploid: 100.00 %

Dip G1: 58.72 % at 58.97

Dip G2: 21.37 % at 112.04

Dip S: 19.91 % G2/G1: 1.90

%CV: 8.95


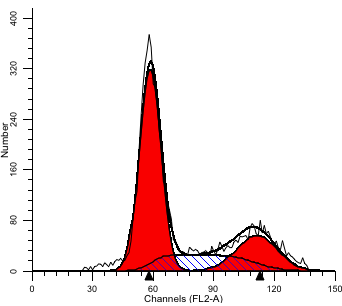


File analyzed: Data.009

Diploid: 100.00 %

Dip G1: 60.57 % at 58.69

Dip G2: 20.15 % at 111.51

Dip S: 19.27 % G2/G1: 1.90

%CV: 9.00

SUDHL4 LV-CDC6


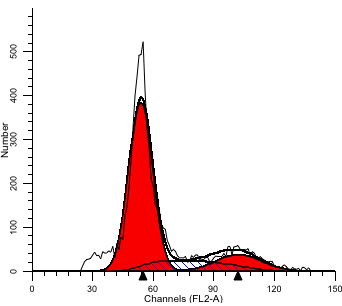


File analyzed: Data.010

Diploid: 100.00 %

Dip G1: 72.32 % at 54.02

Dip G2: 13.11 % at 102.64

Dip S: 14.58 % G2/G1: 1.90

%CV: 10.70


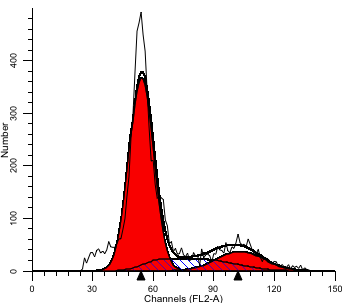


File analyzed: Data.011

Diploid: 100.00 %

Dip G1: 71.43 % at 54.33

Dip G2: 13.67 % at 103.23

Dip S: 14.90 % G2/G1: 1.90

%CV: 11.04


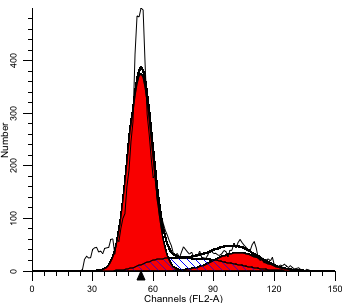


File analyzed: Data.012

Diploid: 100.00 %

Dip G1: 72.10 % at 53.86

Dip G2: 12.65 % at 102.33

Dip S: 15.26 % G2/G1: 1.90

%CV: 10.84

SUDHL4 LV-shCtrl


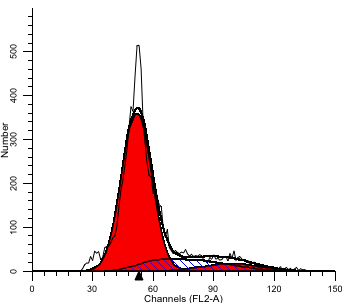


File analyzed: Data.013

Diploid: 100.00 %

Dip G1: 77.75 % at 52.05

Dip G2: 6.63 % at 98.89

Dip S: 15.62 % G2/G1: 1.90

%CV: 13.97


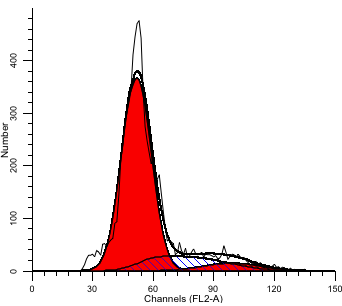


File analyzed: Data.014

Diploid: 100.00 %

Dip G1: 78.02 % at 51.97

Dip G2: 6.08 % at 98.75

Dip S: 15.89 % G2/G1: 1.90

%CV: 13.84


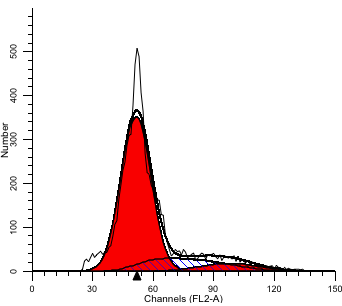


File analyzed: Data.015

Diploid: 100.00 %

Dip G1: 76.65 % at 51.72

Dip G2: 6.93 % at 98.27

Dip S: 16.42 % G2/G1: 1.90

%CV: 14.12

SUDHL4 LV-shCDC6

**The followings are from experiments using OCI-LY7 cells**


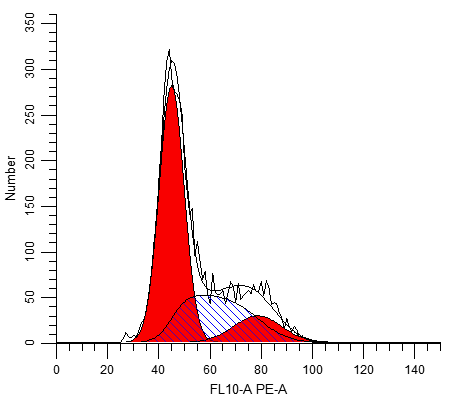


File analyzed: Tube1.fcs

Dip G1: 58.72 % at 44.91

Dip G2: 11.36 % at 87.55

Dip S: 29.92 % G2/G1: 1.95

%CV: 6.79


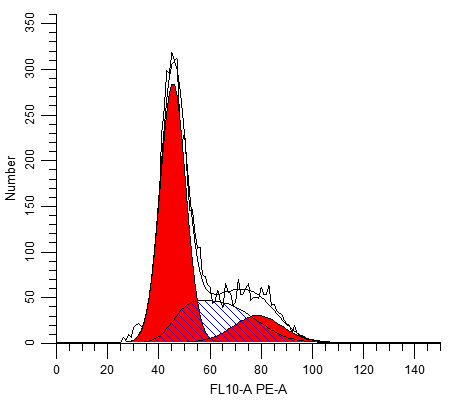


File analyzed: Tube2.fcs

Dip G1: 59.34 % at 45.44

Dip G2: 13.89 % at 87.71

Dip S: 26.77 % G2/G1: 1.93

%CV: 5.93


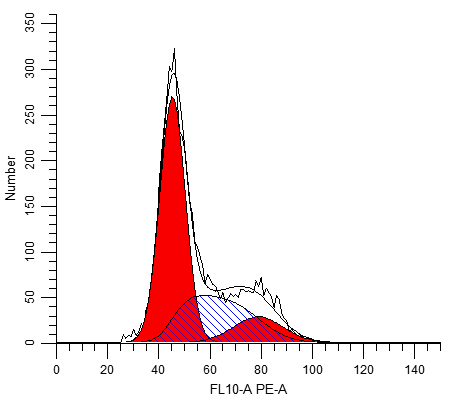


File analyzed: Tube3.fcs

Dip G1: 58.35 % at 45.27

Dip G2: 15.42 % at 87.82

Dip S: 26.23 % G2/G1: 1.94

%CV: 5.98

OCI-Ly7 parental cells


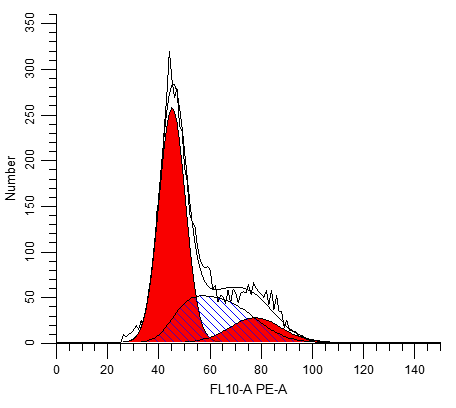


File analyzed: Tube4.fcs

Dip G1: 58.59 % at 45.14

Dip G2: 11.54 % at 86.63

Dip S: 29.87 % G2/G1: 1.92

%CV: 6.42


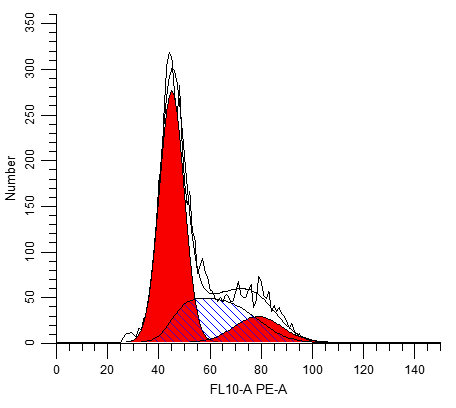


File analyzed: Tube5.fcs

Dip G1: 59.35 % at 44.97

Dip G2: 14.38 % at 87.70

Dip S: 26.27 % G2/G1: 1.95

%CV: 5.73


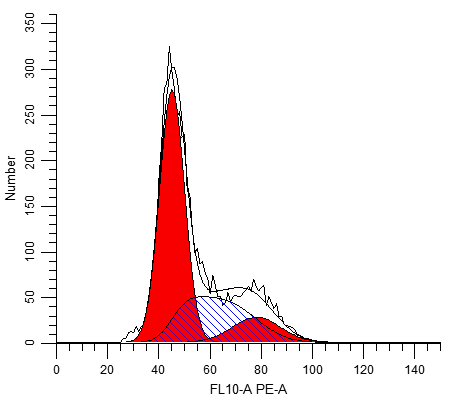


File analyzed: Tube6.fcs

Dip G1: 59.48 % at 45.07

Dip G2: 13.07 % at 86.96

Dip S: 27.45 % G2/G1: 1.93

%CV: 5.82

OCI-LY7 LV-NC


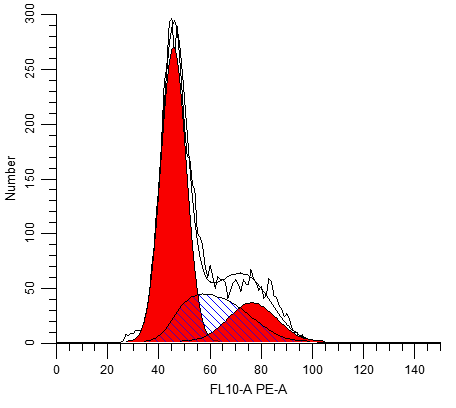


File analyzed: Tube7.fcs

Dip G1: 52.15 % at 45.64

Dip G2: 24.15 % at 90.36

Dip S: 23.70 % G2/G1: 1.98

%CV: 5.87

File analyzed: Tube8.fcs

Dip G1: 51.65 % at 45.51

Dip G2: 26.15 % at 90.12

Dip S: 22.20 % G2/G1: 1.98

%CV: 6.05


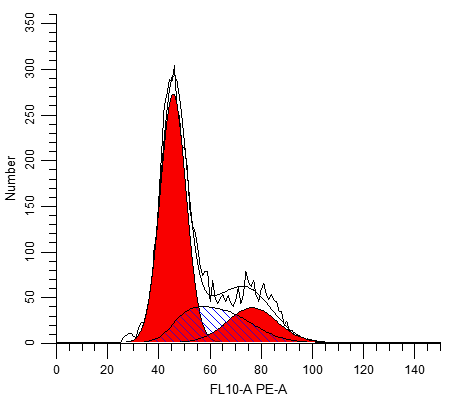


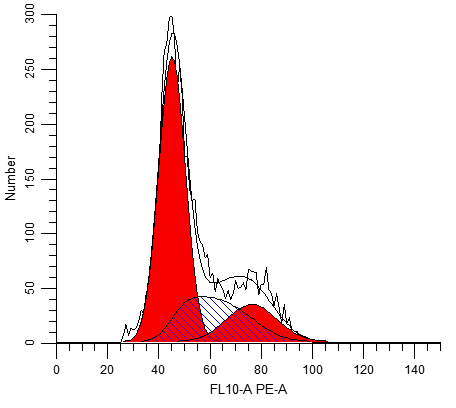


File analyzed: Tube9.fcs

Dip G1: 50.79 % at 45.08

Dip G2: 25.16 % at 88.35

Dip S: 24.05 % G2/G1: 1.96

%CV: 5.61

OCI-LY7 LV-CDC6


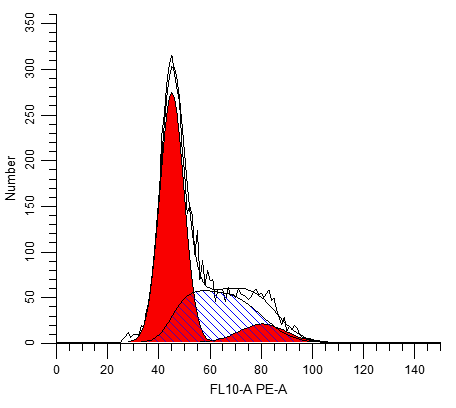


File analyzed: Tube10.fcs

Dip G1: 58.10 % at 45.03

Dip G2: 14.76 % at 88.71

Dip S: 27.14 % G2/G1: 1.97

%CV: 4.47


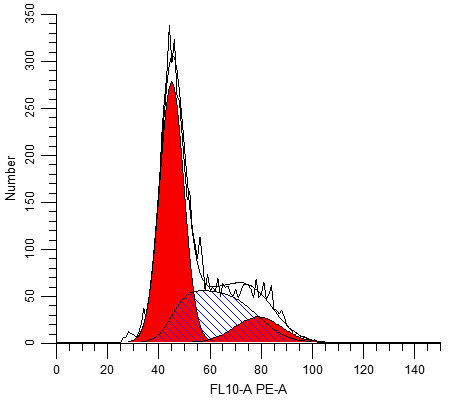


File analyzed: Tube11.fcs

Dip G1: 57.10 % at 44.96

Dip G2: 15.42 % at 87.67

Dip S: 27.48 % G2/G1: 1.95

%CV: 4.48


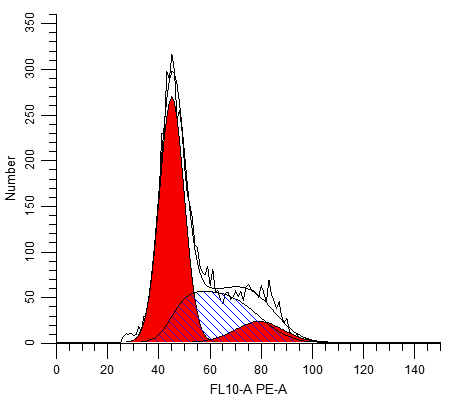


File analyzed: Tube12.fcs

Dip G1: 57.29 % at 44.97

Dip G2: 13.08 % at 88.14

Dip S: 29.63 % G2/G1: 1.96

%CV: 4.76

OCI-LY7 LV-shCtrl


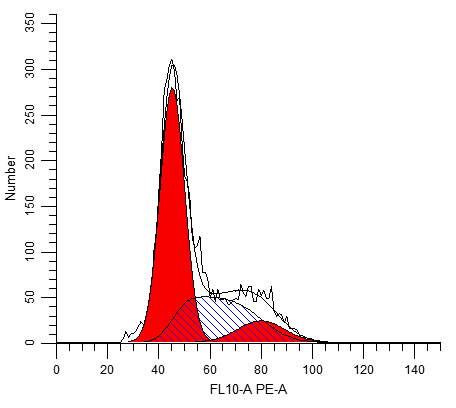


File analyzed: Tube13.fcs

Dip G1: 64.51 % at 45.20

Dip G2: 9.50 % at 89.08

Dip S: 25.99 % G2/G1: 1.97

%CV: 4.56


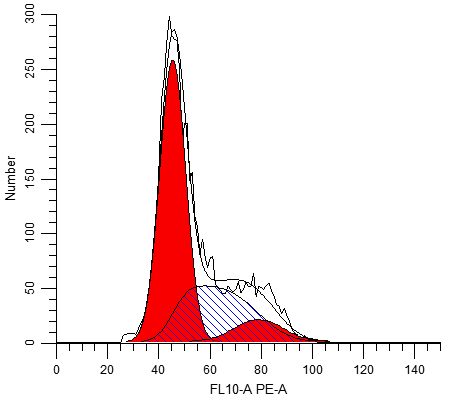


File analyzed: Tube14.fcs

Dip G1: 64.23 % at 45.34

Dip G2: 8.95 % at 87.95

Dip S: 26.81 % G2/G1: 1.94

%CV: 6.17


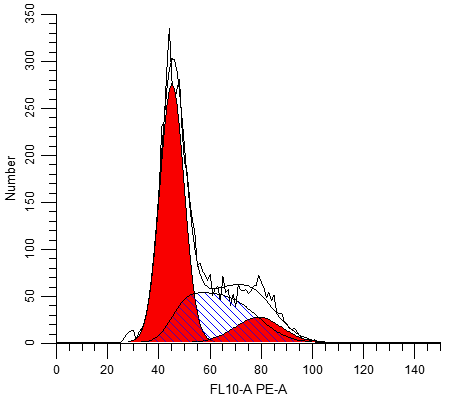


File analyzed: Tube15.fcs

Dip G1: 65.78 % at 45.06

Dip G2: 10.50 % at 87.86

Dip S: 23.72 % G2/G1: 1.95

%CV: 5.52

OCI-LY7 LV-shCDC6

| SUDHL4 |  |  |  |
| --- | --- | --- | --- |
|  |  |  |  |
| **Group** | **G1(%)** | **S(%)** | **G2(%)** |
| Control | 70.44 | 16.74 | 12.81 |
|  | 71.38 | 16.02 | 12.6 |
|  | 70.87 | 15.87 | 13.25 |
| LV-NC | 71.92 | 17.09 | 10.99 |
|  | 72.60 | 14.84 | 12.56 |
|  | 71.15 | 15.12 | 13.73 |
| LV-CDC6 | 59.86 | 19.09 | 21.05 |
|  | 58.72 | 19.91 | 21.37 |
|  | 60.57 | 19.27 | 20.15 |
| LV-sh | 72.32 | 14.58 | 13.11 |
|  | 71.43 | 14.90 | 13.67 |
|  | 72.10 | 15.26 | 12.65 |
| LV-shCDC6 | 77.75 | 15.62 | 6.63 |
|  | 78.02 | 15.89 | 6.08 |
|  | 76.65 | 16.42 | 6.93 |

| OCI-LY7 |  |  |  |
| --- | --- | --- | --- |
|  |  |  |  |
| Group | G1(%) | S(%) | G2(%) |
| CON | 58.72 | 29.92 | 11.36 |
|  | 59.34 | 26.77 | 13.89 |
|  | 58.35 | 26.23 | 15.42 |
| LV-NC | 58.59 | 29.87 | 11.54 |
|  | 59.35 | 26.27 | 14.38 |
|  | 59.48 | 27.45 | 13.07 |
| LV-CDC6 | 52.15 | 23.7 | 24.15 |
|  | 51.65 | 22.2 | 26.15 |
|  | 50.79 | 24.05 | 25.16 |
| LV-shRNA | 58.1 | 27.14 | 14.76 |
|  | 57.1 | 27.48 | 15.42 |
|  | 57.29 | 29.63 | 13.08 |
| LV-shCDC6 | 64.51 | 25.99 | 9.5 |
|  | 64.23 | 26.82 | 8.95 |
|  | 65.78 | 23.72 | 10.5 |
