# Supplementary material for: CDC6, a key replication licensing factor, is overexpressed and confers poor prognosis in diffuse large B-cell lymphoma
Source: BMC Cancer. 2023 Oct 13;23:978. doi: 10.1186/s12885-023-11186-6 (PMC10571299; doi:10.1186/s12885-023-11186-6)
Supplement: Supplementary file 5 — Supplementary Material 5 [file 12885_2023_11186_MOESM5_ESM.docx]

**SUDHL4 control cells 24h**

SUDHL4 parental cells

**SUDHL4 LV-NC 24h**

**SUDHL4 LV-CDC6 24h**

**SUDHL4 LV-shCtrl 24h**

Note: a corrupted image is not shown in this group.

**SUDHL4 LV-shCDC6 24h**

**SUDHL4 cells 48h**

**SUDHL4 LV-NC 48h**

**SUDHL4 LV-CDC6 48h**

**SUDHL4 LV-shCtrl 48h**

**SUDHL4 LV-shCDC6 48h**

**SUDHL4 control cells 72h**

**SUDHL4 LV-NC 72h**

**SUDHL4 LV-CDC6 72h**

**SUDHL4 LV-shCtrl 72h**

**SUDHL4 LV-shCDC6 72h**

|  | **Group** | **UL(%)** | **UR(%)** | **LL(%)** | **LR(%)** | **Apoptosis(%)** |
| --- | --- | --- | --- | --- | --- | --- |
| 24 h | SUDHL4 | 0.92 | 1.64 | 94.74 | 2.69 | 4.33 |
|  |  | 1.10 | 1.48 | 94.35 | 3.06 | 4.54 |
|  |  | 1.02 | 1.52 | 94.91 | 2.55 | 4.07 |
|  | LV-NC | 0.94 | 1.45 | 95.15 | 2.45 | 3.90 |
|  |  | 1.23 | 1.35 | 94.81 | 2.61 | 3.96 |
|  |  | 1.18 | 1.21 | 94.94 | 2.67 | 3.88 |
|  | LV-CDC6 | 1.25 | 1.37 | 95.14 | 2.24 | 3.61 |
|  |  | 1.21 | 1.46 | 94.84 | 2.5 | 3.96 |
|  |  | 1.35 | 1.64 | 94.63 | 2.38 | 4.02 |
|  | LV-shCTRL | 1.43 | 1.70 | 94.42 | 2.45 | 4.15 |
|  |  | 1.23 | 1.61 | 94.54 | 2.62 | 4.23 |
|  |  | 1.11 | 1.60 | 94.86 | 2.43 | 4.03 |
|  | LV-shCDC6 | 0.44 | 5.48 | 84.96 | 9.11 | 14.59 |
|  |  | 0.32 | 5.75 | 84.88 | 9.05 | 14.80 |
|  |  | 0.42 | 6.46 | 85.63 | 7.49 | 13.95 |
| 48 h | SUDHL4 | 1.01 | 1.50 | 94.71 | 2.77 | 4.27 |
|  |  | 1.34 | 1.66 | 94.56 | 2.44 | 4.10 |
|  |  | 1.28 | 1.28 | 95.17 | 2.27 | 3.55 |
|  | LV-NC | 1.03 | 1.84 | 95.04 | 2.1 | 3.94 |
|  |  | 1.57 | 1.77 | 94.31 | 2.35 | 4.12 |
|  |  | 1.18 | 1.81 | 94.32 | 2.69 | 4.50 |
|  | LV-CDC6 | 1.50 | 2.10 | 94.13 | 2.27 | 4.37 |
|  |  | 1.47 | 1.98 | 94.15 | 2.4 | 4.38 |
|  |  | 1.49 | 2.25 | 93.94 | 2.33 | 4.58 |
|  | LV-shCTRL | 1.60 | 1.98 | 93.99 | 2.43 | 4.41 |
|  |  | 1.57 | 1.78 | 94.6 | 2.05 | 3.83 |
|  |  | 1.41 | 2.28 | 94.06 | 2.25 | 4.53 |
|  | LV-shCDC6 | 0.62 | 8.38 | 78.99 | 12.01 | 20.39 |
|  |  | 0.67 | 8.55 | 78.23 | 12.55 | 21.10 |
|  |  | 0.68 | 9.11 | 78.41 | 11.8 | 20.91 |
| 72 h | SUDHL4 | 1.54 | 2.15 | 93.81 | 2.5 | 4.65 |
|  |  | 1.46 | 2.12 | 94.2 | 2.22 | 4.34 |
|  |  | 1.57 | 1.94 | 94.09 | 2.39 | 4.33 |
|  | LV-NC | 0.67 | 1.79 | 94.64 | 2.9 | 4.69 |
|  |  | 0.67 | 1.77 | 94.7 | 2.86 | 4.63 |
|  |  | 0.86 | 1.70 | 94.18 | 3.26 | 4.96 |
|  | LV-CDC6 | 0.69 | 1.83 | 94.43 | 3.05 | 4.88 |
|  |  | 0.82 | 1.75 | 94.33 | 3.1 | 4.85 |
|  |  | 0.86 | 1.66 | 94.41 | 3.08 | 4.74 |
|  | LV-shCTRL | 0.67 | 1.87 | 94.81 | 2.65 | 4.52 |
|  |  | 0.63 | 1.86 | 94.67 | 2.85 | 4.71 |
|  |  | 0.92 | 1.69 | 94.9 | 2.49 | 4.18 |
|  | LV-shCDC6 | 0.82 | 8.73 | 69.32 | 21.14 | 29.87 |
|  |  | 0.54 | 8.36 | 71.39 | 19.71 | 28.07 |
|  |  | 1.16 | 9.06 | 67.60 | 22.18 | 31.24 |

**OCI-LY7 cell apoptosis 24h**


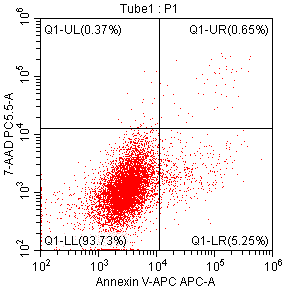

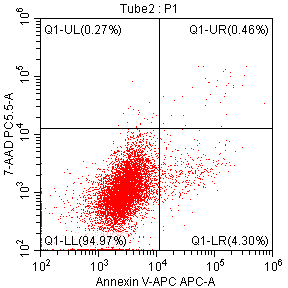


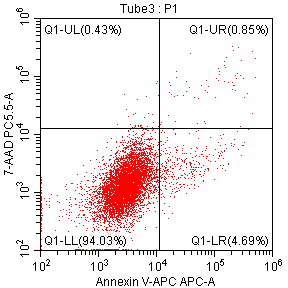


**OCI-LY7 controls 24h**

**OCI-LY7 LV-NC 24h**


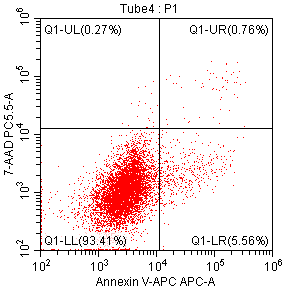

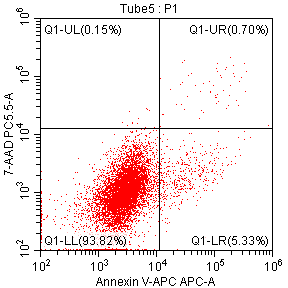


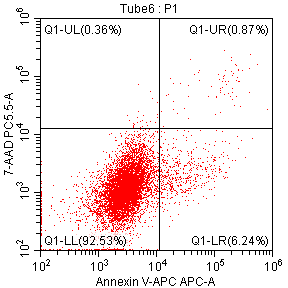


**OCI-LY7 LV-CDC6 24h**


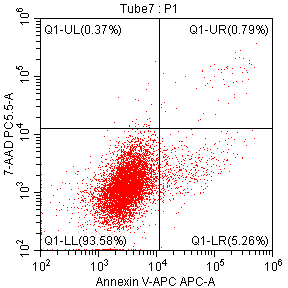

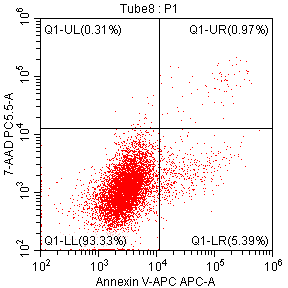


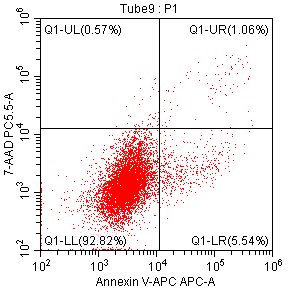


**OCI-LY7 LV-shCtrl 24h**


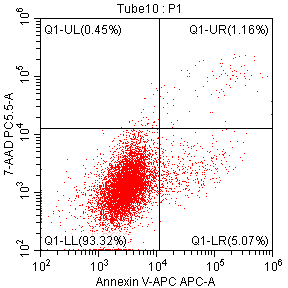

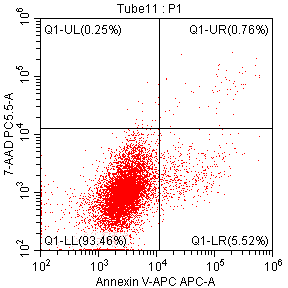

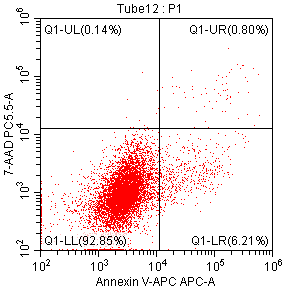


**OCI-LY7 LV-shCDC6 24h**


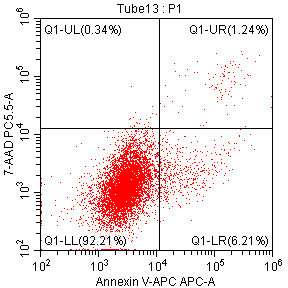

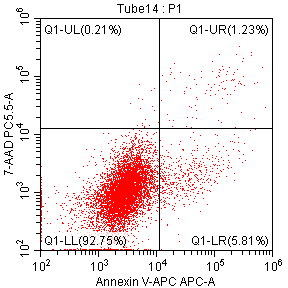

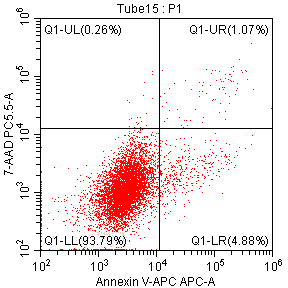


**OCI-LY7 cells, 48h**


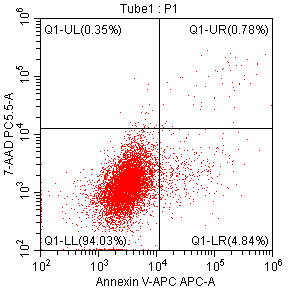

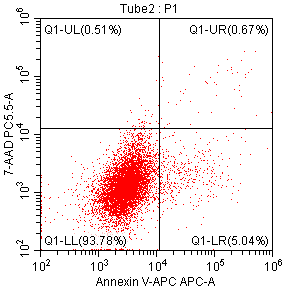


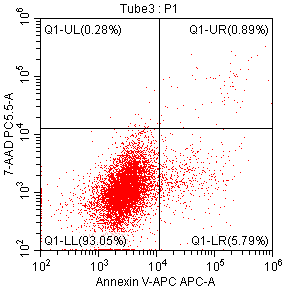


**OCI-LY7 LV-NC, 48h**


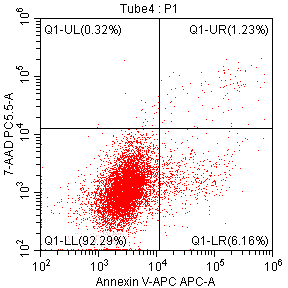

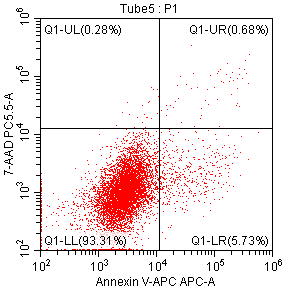


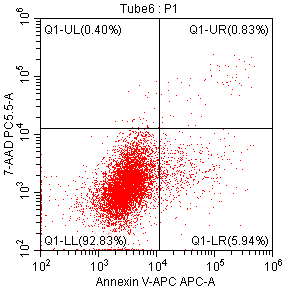


**OCI-LY7 LV-CDC6, 48h**


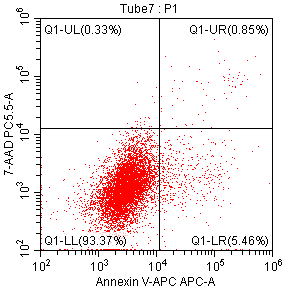

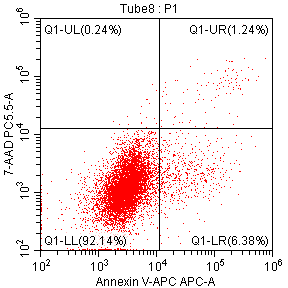


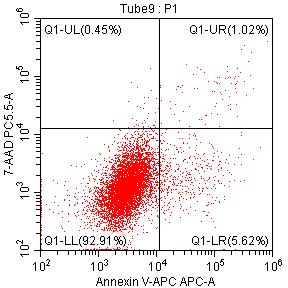


**OCI-LY7 LV-shCtrl, 48h**


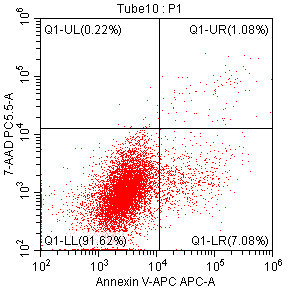

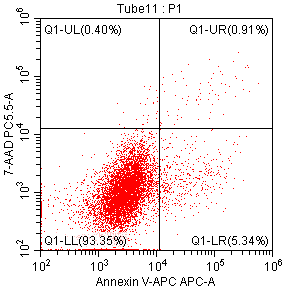


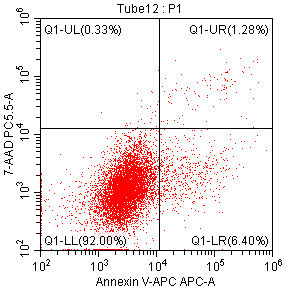


**OCI-LY7 LV-shCDC6, 48h**


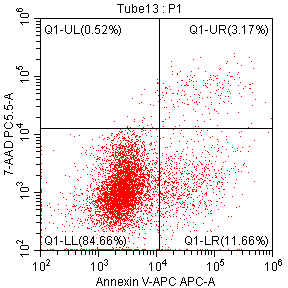

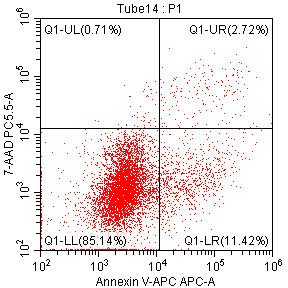

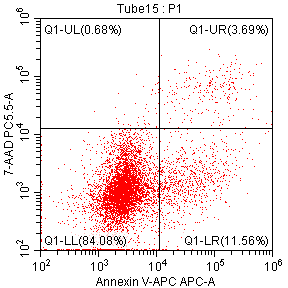


**OCI-LY7 cells control 72h**


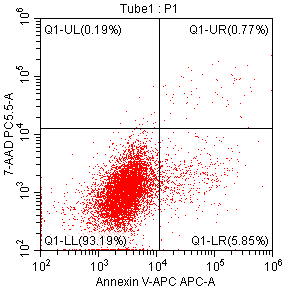

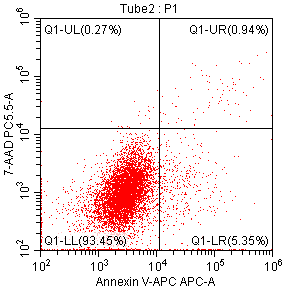


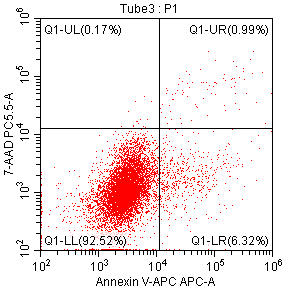


**OCI-LY7 LV-NC 72h**


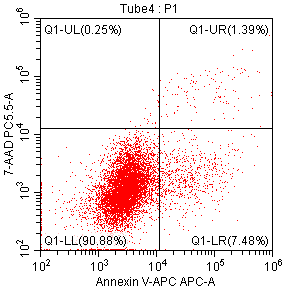

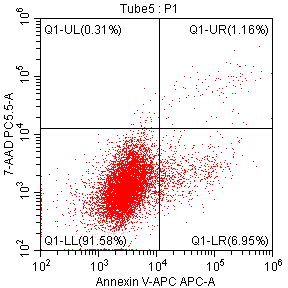


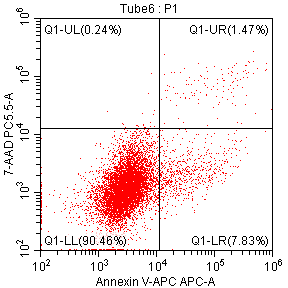


**OCI-LY7 LV-CDC6 72h**


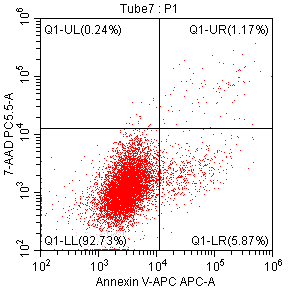

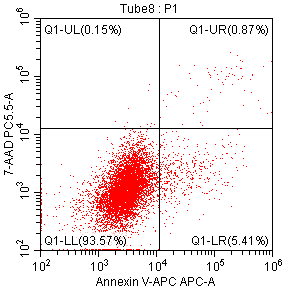


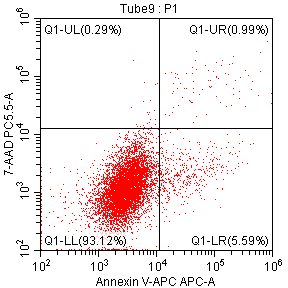


**OCI-LY7 LV-shCtrl 72h**


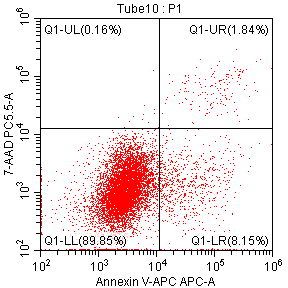

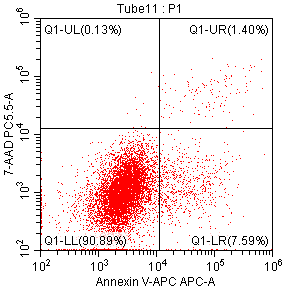


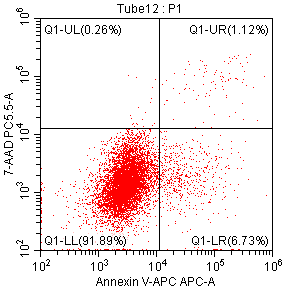


**OCI-LY7 LV-shCDC6 72h**


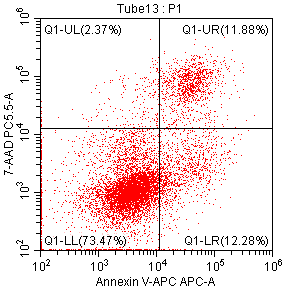

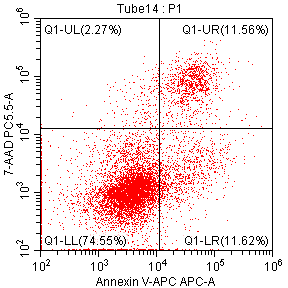

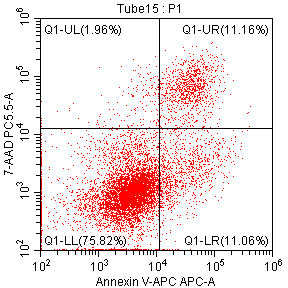


| OCI-LY7 | **Group** | **UL(%)** | **UR(%)** | **LL(%)** | **LR(%)** | **Apoptosis(%)** |
| --- | --- | --- | --- | --- | --- | --- |
|  | CON | 0.37 | 0.65 | 93.73 | 5.25 | 5.90 |
| 24h |  | 0.27 | 0.46 | 94.97 | 4.30 | 4.76 |
|  |  | 0.43 | 0.85 | 94.03 | 4.69 | 5.54 |
|  | LV-NC | 0.27 | 0.76 | 93.41 | 5.56 | 6.32 |
|  |  | 0.15 | 0.70 | 93.82 | 5.33 | 6.03 |
|  |  | 0.36 | 0.87 | 92.53 | 6.24 | 7.11 |
|  | LV-CDC6 | 0.37 | 0.79 | 93.58 | 5.26 | 6.05 |
|  |  | 0.31 | 0.97 | 93.33 | 5.39 | 6.36 |
|  |  | 0.57 | 1.06 | 92.82 | 5.54 | 6.60 |
|  | LV-shCtrl | 0.45 | 1.16 | 93.32 | 5.07 | 6.23 |
|  |  | 0.25 | 0.76 | 93.46 | 5.52 | 6.28 |
|  |  | 0.14 | 0.80 | 92.85 | 6.21 | 7.01 |
|  | LV-shCDC6 | 0.34 | 1.24 | 92.21 | 6.21 | 7.45 |
|  |  | 0.21 | 1.23 | 92.75 | 5.81 | 7.04 |
|  |  | 0.26 | 1.07 | 93.79 | 4.88 | 5.95 |
|  |  |  |  |  |  |  |
| OCI-LY7 | **Group** | **UL(%)** | **UR(%)** | **LL(%)** | **LR(%)** | **Apoptosis(%)** |
|  | CON | 0.35 | 0.78 | 94.03 | 4.84 | 5.62 |
| 48h |  | 0.51 | 0.67 | 93.78 | 5.04 | 5.71 |
|  |  | 0.28 | 0.89 | 93.05 | 5.79 | 6.68 |
|  | LV-NC | 0.32 | 1.23 | 92.29 | 6.16 | 7.39 |
|  |  | 0.28 | 0.68 | 93.31 | 5.73 | 6.41 |
|  |  | 0.40 | 0.83 | 92.83 | 5.94 | 6.77 |
|  | LV-CDC6 | 0.33 | 0.85 | 93.37 | 5.46 | 6.31 |
|  |  | 0.24 | 1.24 | 92.14 | 6.38 | 7.62 |
|  |  | 0.45 | 1.02 | 92.91 | 5.62 | 6.64 |
|  | LV-shCtrl | 0.22 | 1.08 | 91.62 | 7.08 | 8.16 |
|  |  | 0.40 | 0.91 | 93.35 | 5.34 | 6.25 |
|  |  | 0.33 | 1.28 | 92.00 | 6.40 | 7.68 |
|  | LV-shCDC6 | 0.52 | 3.17 | 84.66 | 11.66 | 14.83 |
|  |  | 0.71 | 2.72 | 85.14 | 11.42 | 14.14 |
|  |  | 0.68 | 3.69 | 84.08 | 11.56 | 15.25 |
|  |  |  |  |  |  |  |
| OCI-LY7 | **Group** | **UL(%)** | **UR(%)** | **LL(%)** | **LR(%)** | **Apoptosis(%)** |
|  | CON | 0.19 | 0.77 | 93.19 | 5.85 | 6.62 |
| 72h |  | 0.27 | 0.94 | 93.45 | 5.35 | 6.29 |
|  |  | 0.17 | 0.99 | 92.52 | 6.32 | 7.31 |
|  | LV-NC | 0.25 | 1.39 | 90.88 | 7.48 | 8.87 |
|  |  | 0.31 | 1.16 | 91.58 | 6.95 | 8.11 |
|  |  | 0.24 | 1.47 | 90.46 | 7.83 | 9.30 |
|  | LV-CDC6 | 0.24 | 1.17 | 92.73 | 5.87 | 7.04 |
|  |  | 0.15 | 0.87 | 93.57 | 5.41 | 6.28 |
|  |  | 0.29 | 0.99 | 93.12 | 5.59 | 6.58 |
|  | LV-shCtrl | 0.16 | 1.84 | 89.85 | 8.15 | 9.99 |
|  |  | 0.13 | 1.40 | 90.89 | 7.59 | 8.99 |
|  |  | 0.26 | 1.12 | 91.89 | 6.73 | 7.85 |
|  | LV-shCDC6 | 2.37 | 11.88 | 73.47 | 12.28 | 24.16 |
|  |  | 2.27 | 11.56 | 74.55 | 11.62 | 23.18 |
|  |  | 1.96 | 11.16 | 75.82 | 11.06 | 22.22 |
