# Supplementary material for: CDC6, a key replication licensing factor, is overexpressed and confers poor prognosis in diffuse large B-cell lymphoma
Source: BMC Cancer. 2023 Oct 13;23:978. doi: 10.1186/s12885-023-11186-6 (PMC10571299; doi:10.1186/s12885-023-11186-6)
Supplement: Supplementary file 6 — Supplementary Material 6 [file 12885_2023_11186_MOESM6_ESM.docx]

| **DLBCL sample information** | | | | | | | | |
| --- | --- | --- | --- | --- | --- | --- | --- | --- |
| patient no. | Pathol. | Gender  Male 0  Female1 | Age | IPI  score | Stage | Hans | CDC6 expression score | CDC6 positivity  <6 negative（0）；  >=6 positve ( 1） |
| 10 | 12-07383 | 0 | 66 | 1 | IA | GCB | 3 | 0 |
| 9 | 12-18539 | 0 | 59 | 1 | IIA | GCB | 2 | 0 |
| 8 | 12-19225 | 0 | 77 | 1 | IA | non-GCB | 6 | 1 |
| 7 | 12-27057 | 0 | 19 | 2 | IVB | non-GCB | 0 | 0 |
| 6 | 13-01846 | 0 | 49 | 2 | IVB | non-GCB | 6 | 1 |
| 15 | 13-02573 | 1 | 79 | 3 | IVA | non-GCB | 4 | 0 |
| 12 | 13-03445 | 1 | 75 | 4 | IVB | non-GCB | 6 | 1 |
| 13 | 13-10710 | 1 | 65 | 4 | IVB | non-GCB | 4 | 0 |
| 14 | 13-13850 | 0 | 57 | 1 | IIIA | GCB | 3 | 0 |
| 11 | 13-14262 | 0 | 74 | 1 | IIA | GCB | 6 | 1 |
| 16 | 13-15239 | 1 | 52 | 0 | IA | GCB | 6 | 1 |
| 17 | 13-26037 | 1 | 49 | 1 | IIIA | non-GCB | 6 | 1 |
| 18 | 13-30264 | 0 | 62 | 1 | IIA | GCB | 6 | 1 |
| 19 | 14-00623 | 0 | 60 | 1 | IA | non-GCB | 6 | 1 |
| 20 | 14-00745 | 1 | 47 | 1 | IA | non-GCB | 6 | 1 |
| 21 | 14-11786 | 0 | 64 | 3 | IVA | non-GCB | 6 | 1 |
| 22 | 14-14512 | 0 | 78 | 2 | IA | GCB | 3 | 0 |
| 23 | 14-18373 | 0 | 64 | 3 | IVA | GCB | 3 | 0 |
| 5 | 14-21904 | 1 | 49 | 0 | IA | non-GCB | 6 | 1 |
| 4 | 14-22536 | 1 | 64 | 2 | IIIA | GCB | 3 | 0 |
| 3 | 14-31381 | 0 | 86 | 3 | IIIB | non-GCB | 9 | 1 |
| 2 | 15-00889 | 0 | 70 | 4 | IVB | non-GCB | 9 | 1 |
| 1 | 15-06381 | 0 | 65 | 3 | IVB | non-GCB | 4 | 0 |
| 42 | 15-16434 | 0 | 72 | 2 | IA | GCB | 4 | 0 |
| 41 | 15-23611 | 1 | 68 | 2 | IIA | GCB | 6 | 1 |
| 40 | 15-24504 | 0 | 78 | 2 | IIA | non-GCB | 6 | 1 |
| 39 | 15-30173 | 0 | 58 | 1 | IIA | non-GCB | 4 | 0 |
| 38 | 15-33863 | 0 | 66 | 1 | IIA | GCB | 2 | 0 |
| 47 | 15-35139 | 1 | 50 | 2 | IIIA | GCB | 4 | 0 |
| 46 | 16-10435 | 0 | 73 | 1 | IA | non-GCB | 0 | 0 |
| 44 | 16-12072 | 0 | 58 | 0 | IIA | non-GCB | 6 | 1 |
| 45 | 16-14030 | 1 | 68 | 2 | IVB | non-GCB | 6 | 1 |
| 43 | 16-16481 | 1 | 53 | 2 | IVA | non-GCB | 6 | 1 |
| 26 | 16-20491 | 1 | 78 | 1 | IIA | non-GCB | 6 | 1 |
| 28 | 16-22419 | 0 | 75 | 3 | IIIA | non-GCB | 9 | 1 |
| 63 | 16-34165 | 1 | 52 | 0 | IA | GCB | 1 | 0 |
| 29 | 16-40044 | 0 | 61 | 2 | IV A | non-GCB | 6 | 1 |
| 30 | 16-40084 | 0 | 51 | 1 | IA | GCB | 3 | 0 |
| 31 | 17-13686 | 0 | 68 | 1 | IIA | non-GCB | 0 | 0 |
| 25 | 17-15536 | 1 | 53 | 0 | IIA/ivA? | GCB | 2 | 0 |
| 27 | 17-17485 | 0 | 67 | 3 | ivA | non-GCB | 4 | 0 |
| 24 | 17-33712 | 0 | 44 | 0 | IA | non-GCB | 4 | 0 |
| 69 | 18-06292 | 0 | 65 | 2 | IIIA | non-GCB | 6 | 1 |
| 64 | 18-08825 | 1 | 76 | 1 | IIA | GCB | 6 | 1 |
| A | 18-09082 | 1 | 60 | 3 | IIIA | non-GCB | 6 | 1 |
| 65 | 18-11264 | 0 | 70 | 2 | IIA | non-GCB | 6 | 1 |
| 66 | 18-17085 | 0 | 71 | 3 | IVA | non-GCB | 6 | 1 |
| 68 | 18-18160 | 1 | 76 | 3 | IIIB | non-GCB | 6 | 1 |
| 70 | 18-19789 | 1 | 80 | 1 | IA | non-GCB | 9 | 1 |
| 71 | 18-19823 | 1 | 80 | 1 | IIA | GCB | 6 | 1 |
| 72 | 18-27587 | 0 | 56 | 1 | IA | GCB | 0 | 0 |
| 73 | 18-35402 | 1 | 63 | 2 | IIIB | non-GCB | 6 | 1 |
| 74 | 18-36397 | 0 | 74 | 3 | IVB | non-GCB | 4 | 0 |
| 75 | 18-37012 | 0 | 63 | 3 | IVA | non-GCB | 6 | 1 |
| 76 | 18-37560 | 1 | 68 | 1 | IA | GCB | 2 | 0 |
| 77 | 18-39136 | 0 | 75 | 2 | IIIA | non-GCB | 6 | 1 |
| G | 18-9588G | 0 | 62 | 1 | IA | GCB | 4 | 0 |
| F | 18-35826F | 1 | 54 | 0 | IA | non-GCB | 6 | 1 |
| 79 | 17-18856 | 1 | 59 | 0 | IA | non-GCB | 4 | 0 |
| 78 | 18-25366 | 1 | 70 | 2 | IA | GCB | 6 | 1 |

| **Lymph node hyperplasia (as control)** | | | | |
| --- | --- | --- | --- | --- |
| patent no | Pathol. No | Gender  (Male 0  Female 1) | CDC6 expression score | CDC6 expression positivity  <6 negative（0）；>=6 positve (1） |
| 32 | 18-12532 | 1 | 4 | 0 |
| 48 | 18-13964 | 0 | 2 | 0 |
| 49 | 18-19622 | 0 | 4 | 0 |
| 37 | 18-20509 | 0 | 4 | 0 |
| 36 | 18-21458 | 0 | 4 | 0 |
| 35 | 18-22350 | 0 | 0 | 0 |
| 34 | 18-23328 | 1 | 9 | 1 |
| 54 | 18-31470 | 1 | 0 | 0 |
| 55 | 18-35104 | 0 | 4 | 0 |
| 56 | 18-41373 | 1 | 4 | 0 |
| 57 | 18-41541 | 1 | 6 | 1 |
| 50 | 18-42484 | 0 | 6 | 1 |
| 51 | 18-42899 | 1 | 4 | 0 |
| 61 | S19-00607 | 0 | 6 | 1 |
| 62 | S19-00904 | 1 | 6 | 1 |
| 52 | 19-10037 | 1 | 4 | 0 |
| 53 | S19-00034 | 0 | 0 | 0 |
| 58 | S19-00247 | 0 | 2 | 0 |
| 59 | S19-00458 | 0 | 4 | 0 |
| 60 | S19-00467 | 0 | 4 | 0 |

Immunohistochemistry (IHC): DLBCL or LN hyperplasia specimens were fixed in 10% neutral buffered formalin solution, embedded in paraffin, serially sectioned at 4μm, and then stained with CDC6 monoclonal antibody (ab109315) by the EnVision Detection System K5007 (Dako, Denmark). To quantity CDC6 expression, five high-power fields (x200) were randomly selected from each section, and 500 cells were counted and calculate the immunoreactive score ranging from 0 to 12, with <6 being negative expression and ≥6 being positive expression.
