# Supplementary material for: CDC6, a key replication licensing factor, is overexpressed and confers poor prognosis in diffuse large B-cell lymphoma
Source: BMC Cancer. 2023 Oct 13;23:978. doi: 10.1186/s12885-023-11186-6 (PMC10571299; doi:10.1186/s12885-023-11186-6)
Supplement: Supplementary file 7 — Supplementary Material 7 [file 12885_2023_11186_MOESM7_ESM.docx]

**Supplemental Figure 1**

**Supplemental Figure 1, The role of CDC6 in cell cycle regulation**

In GSE10846 (n=414) , 233 cases were treated with R-CHOP regimen and the others were treated with CHOP regimen. We analyzed the association between CDC6 expression and patients’ prognosis in the R-CHOP subgroup. The result showed that patients with high CDC6 expression tend to have a poor overall survival than those with low CDC6 expression, although the difference was not statistically significant (p=0.104).


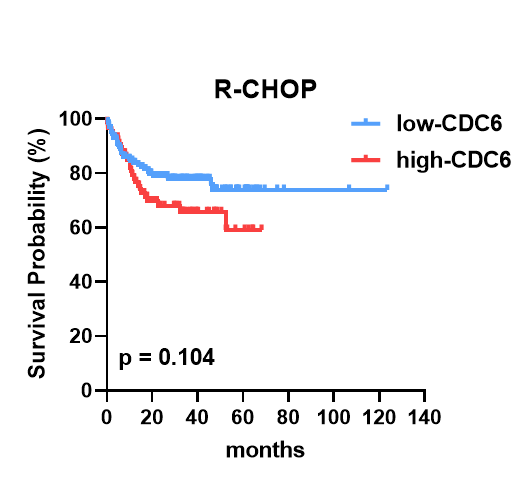


**Supplemental Figure 2,** OS stratified by the status of CDC6 expression in patients receiving R-CHOP

As a result, CDC6, IPI score, stage, HB, LDH, and classification were significantly associated with OS in univariate analysis (all P＜0.05). Furthermore, the multivariate analysis showed that HR values of CDC6 (positive vs. negative), HB (≥100 vs.＜100), and classification (Non-GCB vs. GCB) were 3.098 (P = 0.038), 4.042 (P = 0.002), and 3.847(P = 0.049), respectively. These results suggested that CDC6 expression was an independent risk factor affecting the prognosis of DLBCL patients.

**Supplementary** **Table 1**. Univariate and multivariate Cox regression analysis of OS in DLBCL patients.

| Prognostic parameter | Univariate analysis | P-value | Multivariate analysis | P-value |
| --- | --- | --- | --- | --- |
|  | HR (95%CI) |  | HR (95%CI) |  |
| CDC6 (positive vs. negative) | 3.281 (1.296 -8.311) | **0.012** | 3.098 (1.064-9.020) | **0.038** |
| Age (≥60 vs.＜60) | 1.398 (0.554 - 3.524) | 0.478 | - | - |
| Gender (female vs. male) | 1.426 (0.637 - 3.193) | 0.388 | - | - |
| IPI score (3-5 vs. 0-2) | 2.630 (1.129 - 6.127) | **0.025** | 1.245 (0.409-3.787) | 0.699 |
| Stage (III-VI vs. I-II) | 3.345 (1.412 - 7.924) | **0.006** | 2.368 (0.735-7.628) | 0.149 |
| HB (≥100 vs.＜100) | 4.196 (1.851 - 9.513) | **0.001** | 4.042 (1.679-9.730) | **0.002** |
| LDH (≥245 vs.＜245) | 2.916 (1.256 - 6.769) | **0.013** | 1.378 (0.486-3.910) | 0.546 |
| Site (Extranodal vs. Nodal) | 0.780 (0.349 - 1.742) | 0.544 | - | **-** |
| Classification (Non-GCB vs. GCB) | 7.584 (2.168 - 26.529) | **0.002** | 3.847 (1.001-14.787) | **0.049** |

HB: Hemoglobin (g/L); LDH: Lactate Dehydrogenase (U/L); HR: hazard ratio; CI: credible interval.
