# Supplementary material for: CDC6, a key replication licensing factor, is overexpressed and confers poor prognosis in diffuse large B-cell lymphoma
Source: BMC Cancer. 2023 Oct 13;23:978. doi: 10.1186/s12885-023-11186-6 (PMC10571299; doi:10.1186/s12885-023-11186-6)
Supplement: Supplementary file 1 — Supplementary Material 1 [file 12885_2023_11186_MOESM1_ESM.docx]

NOTE: We repeated the WBs for SUDHL-4 cells during the revision, and the result was consistent, so we updated the SUDHL-4 data. For WB experiments on SUDHL-4 and OCI-LY7 cells, we now provide both the original full-length and cropped blots (red boxes denoted) in the supplementary file. We did WBs in duplicate; the samples were loaded in parallel with a protein marker. However, the full membrane edges are not clear enough possibly due to inappropriate set-up of the imaging system. We have made some image adjustments to show as much as possible where the marker goes, which may help define the location of membrane edges.

**SUDHL4 GAPDH1**

Ctrl	NC CDC6 shCtrl shCDC6 Ctrl	NC CDC6 shCtrl shCDC6

 exposure adjusted

**SUDHL4, CDC6, 62KD, run in 10% gel**

Ctrl	NC CDC6 shCtrl shCDC6 Ctrl	NC CDC6 shCtrl shCDC6

long exposure

Marker (KD)

118

65

47

36

**SUDHL4, caspase3, 32KD, run in 13% gel**

Ctrl	NC CDC6 shCtrl shCDC6 Ctrl	NC CDC6 shCtrl shCDC6

Long expo.

Marker (KD)

118

65

47

36

26

SUDHL4, bcl-2, 26KD, run in 13% gel

Ctrl	NC CDC6 shCtrl shCDC6 Ctrl	NC CDC6 shCtrl shCDC6

Marker (KD)

118

65

47

36

26

**SUDHL4-Bax, 19KD, run in 13% gel**

Ctrl	NC CDC6 shCtrl shCDC6 Ctrl	NC CDC6 shCtrl shCDC6

Marker (KD)

118

65

47

36

26

**SUDHL4, GAPDH2, run in 10% gel**

Ctrl	NC CDC6 shCtrl shCDC6 Ctrl	NC CDC6 shCtrl shCDC6

Long exposure

Marker (KD)

118

65

47

36

**SUDHL4, INK4, 14KD, run in 10% gel**

Ctrl	NC CDC6 shCtrl shCDC6 Ctrl	NC CDC6 shCtrl shCDC6

Marker (KD)

118

65

47

36

26

**SUDHL4-E-caderherin, 120KD, run in 10% gel**

Ctrl	NC CDC6 shCtrl shCDC6 Ctrl	NC CDC6 shCtrl shCDC6

Marker (KD)

118

65

47

36

**SUDHL4-ATR, 70KD, run in 10% gel**

Ctrl	NC CDC6 shCtrl shCDC6 Ctrl	NC CDC6 shCtrl shCDC6

Marker (KD)

118

65

47

36

**OCI-LY7, GAPDH1, 37KD, run in 10% gel**

Ctrl	NC CDC6 shCtrl shCDC6 Ctrl	NC CDC6 shCtrl shCDC6

Marker (KD)

118

65

47

36

**OCI-LY7, CDC6, 62KD, run in 10% gel**

Ctrl	NC CDC6 shCtrl shCDC6 Ctrl	NC CDC6 shCtrl shCDC6

Marker (KD)

118

65

47

36

26

**OCI-LY7-Caspase3, 32KD, run in 14% gel**

Ctrl	NC CDC6 shCtrl shCDC6 Ctrl	NC CDC6 shCtrl shCDC6

Marker (KD)

118

65

47

36

**OCI-LY7, Bcl2, 26KD, run in 13% gel**

Ctrl	NC CDC6 shCtrl shCDC6 Ctrl	NC CDC6 shCtrl shCDC6

Marker (KD)

118

65

47

36

**OCI-LY7-Bax, 19KD, run in 13% gel**

Ctrl	NC CDC6 shCtrl shCDC6 Ctrl	NC CDC6 shCtrl shCDC6

Marker (KD)

118

65

47

36

26

**OCI-LY7-GAPDH2, 37KD, run in 10% gel**

Ctrl	NC CDC6 shCtrl shCDC6 Ctrl	NC CDC6 shCtrl shCDC6

Marker (KD)

118

65

47

36

26

**OCI-LY7, INK4, 14kd, run in 10% gel**

Ctrl	NC CDC6 shCtrl shCDC6 Ctrl	NC CDC6 shCtrl shCDC6

Marker (KD)

118

65

47

36

**OCI-LY7-E-cadherin, 120kd, run in 10% gel**

Ctrl	NC CDC6 shCtrl shCDC6 Ctrl	NC CDC6 shCtrl shCDC6

Marker (KD)

118

65

47

36

**OCI-LY7-ATR, 70kd, run in 10% gel**

Ctrl	NC CDC6 shCtrl shCDC6 Ctrl	NC CDC6 shCtrl shCDC6

Marker (KD)

118

65

47

36
